# Supplementary material for: Safe and valid? A systematic review of the psychometric properties of culturally adapted depression scales for use among Indigenous populations
Source: Glob Ment Health (Camb). 2023 Sep 14;10:e60. doi: 10.1017/gmh.2023.52 (PMC10579654; doi:10.1017/gmh.2023.52)
Supplement: Yang et al. supplementary material 1 — Yang et al. supplementary material [file S2054425123000523sup001.docx]

**Supplemental 1**

*Systematic search table*

| **PubMed** | **MEDLINE** | **PsycInfo** | **Embase** | **Global Health** | **CINAHL** |
| --- | --- | --- | --- | --- | --- |
| Depression Terms | | | | | |
| “Depressive Disorder, Major*” [MeSH] | Depressive Disorder, Major/ | Exp Major depression/ | Major depression/ | Depression/ | MH "Depression/EP" |
| “Depression*” [MeSH] | Exp Depression/ | Exp “Depression (Emotion)”/ | Exp Depression/ |  |  |
| “Depressive Disorder*” [MeSH] | Depressive disorder/ |  |  |  |  |
| “Depression/epidemiology”[MeSH] | Depression/ep |  | Depression/ep |  |  |
| “Depressive symptoms” [MeSH] | Depressive symptoms.mp. |  | Depressive symptoms.mp. |  |  |
| “Depression, postpartum/diagnosis*” [MeSH] | Exp Depression, postpartum/di | Exp Postpartum Depression/ | Postnatal depression/ |  | MH "Depression, Postpartum" |
| “Stress, Psychological*” [MeSH] | Exp Stress, psychological/ | Exp psychological stress/ | Mental stress/ | Mental stress/ | MH "Stress, Psychological" |
| “Emotions” [MeSH] | Emotions/ | Exp Emotional states/ | Emotion/ | Emotions.mp. | MH "Emotions" |
| “Affective symptoms” [MeSH] | Affective Symptoms/ | Exp Emotional Responses/ |  |  | MH "Affective Symptoms" |
| “Distress, psychological*” [MeSH] | Exp Psychological Distress/ | Exp Distress/ | Distress.mp. | Distress.mp. | "Distress" |
| “Mental Health/ethnology*” [MeSH] | Exp Mental health/eh | Exp Mental Health/ | Mental health/ep | Mental health.mp. | MH "Mental Health" |
| “Mental Disorders/ethnology*” [MeSH] | Exp mental disorders/eh | Exp Mental Disorders/ | Mental disease/ep | Mental disorders.sh. | "Mental health disorder" |
| “Mental Disorders/psychology*” [MeSH] | Exp mental disorders/px |  |  |  |  |
| “Mood disorder” [MeSH] | Mood disorders/ |  |  |  |  |
| Indigenous Terms | | | | | |
| “Indigenous people*” [MeSH] | Exp Indigenous peoples/ | Exp Indigenous Populations/ | Indigenous people/ | Indigenous people/ | MH “Indigenous Peoples” |
| “Health Services, Indigenous*” [MeSH] | Exp Health services, Indigenous/ |  | Indigenous health care |  | MH “Health Services, Indigenous” |
| “Indians, North American*” [MeSH] | Exp Indians, North American/ | Exp American Indians/ | American Indian/ | American Indians/ | MH “Native Americans” |
| “Ethnic groups*” [MeSH] | Exp Ethnic groups/ | Ethnic groups.ti,ab | Ethnic group/ | Ethnic groups/ | MH “Ethnic Groups” |
| “Oceanic ancestry group*” [MeSH] | Exp Oceanic ancestry group/ | Oceanic*.mp. | Oceanic ancestry group/ | Oceanic*.mp. | “Oceania" |
| “Austronesian” [Other Term] | Austronesian.ti,ab | Austronesian.ti,ab | Austronesian people/ | Austronesian.mp. | “Austronesian” |
| “Circumpolar” [Other Term] | Artic Regions/ep | Circumpolar.ti,ab | Artic/ep | Nordic countries.gl. | “Circumpolar” |
| “Alaska natives” [MeSH] | Alaska Natives/px | Exp Alaska Natives/ | Alaska native/ | Alaska.gl. | MH “Eskimos” |
| “Australia/epidemiology” [MeSH] | Exp Australia/ep | Australia*.ti,ab | Indigenous Australia/ | Australia/ | MH “Aboriginal Australians” |
| “Asian continental ancestry group” [MeSH] | Exp Asian continental Ancestry Group/ | Exp Asians/ | Asia/ep | Asia/ | MH “Asia/EH” |
| “Africa*” [MeSH] | Exp African Continental Ancestry Group/ | Exp African Cultural Groups/ | Africa/ep | Africa/ | MH “Africa/EH” |
| “Global Health/ethnology*” [MeSH] | Exp Global health/eh | Exp Global health/ | Global health/ | Global health.mp. | “Global health” |
| “Developing countries*” [MeSH] | Exp Developing Countries/ | Exp Developing Countries/ | Developing country/ | Developing countries/ | MH “Developing countries” |
| “Inuits/psychology*” [MeSH] | Exp Inuits/px | Exp Inuits/ | Inuit/ | Inuit/ | MH “Inuit” |
| “West Indies/ethnology” [MeSH] | West Indies/eh |  | West Indies.mp. | Caribbean/ | MH “West Indies/EH” |
| “South America/ethnology” [MeSH] | South America/eh |  | South America/ep | South America/ | MH “South America” |
| tribal[tiab] | tribal.tw.kf. | tribal.ti,ab. | tribal.ti,ab. | tribal.ti,ab. | “tribal” |
| tribe*[tiab] | tribe*.tw.kf. | tribe*.ti,ab. | tribe*.ti,ab. | tribe*.ti,ab. | “tribe*” |
| pastoral*[tiab] | pastoral*.tw.kf. | pastoral*.ti,ab. | pastoral*.ti,ab. | pastoral*.ti,ab. | “pastoral*” |
| "hunter gatherer"*[tiab] | hunter-gatherer*.tw.kf. | hunter-gatherer*.ti,ab. | hunter-gatherer*.ti,ab. | hunter-gatherer*.ti,ab. | “hunter-gatherer*” |
| "hunter gatherer"*[tiab] | Aborigin*.ti,ab,kf. | aborigin*.ti,ab. | aborigin*.ti,ab. | aborigin*.ti,ab. | “aborigin*” |
| metis[tiab] | Metis.tw.kf. | metis.ti,ab. | metis.ti,ab. | metis.ti,ab. | “metis” |
| autochtone*[tiab] | autochtone*.tw.kf. | autochton*.ti,ab. | autochton*.ti,ab. | autochton*.ti,ab. | “autochton*” |
| Mestizo[tiab] | Mestizo.tw.kf. | mestizo.ti,ab. | mestizo.ti,ab. | mestizo.ti,ab. | “mestizo” |
| Nomadic[tiab] | Nomadic.tw.kf. | nomadic.ti,ab. | nomadic.ti,ab. | nomadic.ti,ab. | “nomadic” |
| Mobs[tiab] | Mobs .tw.kf. | mobs .tw.kf. | mobs .tw.kf. | mobs .tw.kf. | “mobs” |
| "indians, south american"[MeSH Terms] | exp Indians, South American/ | south american indian.ti,ab. | south american indian.ti,ab. | south american indian.ti,ab. | “south american indian” |
| Amerindian*[tiab] | Amerindian*.tw.kf. | Amerindian*.ti,ab. | Amerindian*.ti,ab. | Amerindian*.ti,ab. | “Amerindian*” |
| Indigenas[tiab] | Indigenas.ti,ab,kf. | indigenas.ti,ab. | indigenas.ti,ab. | indigenas.ti,ab. | “indigenas” |
| Nativos Americanos[tiab] | Nativos americanos.tw.kf. | Nativos americanos.ti,ab. | Nativos americanos.ti,ab. | Nativos americanos.ti,ab. | “Nativos Americanos” |
| Small ethnic populations[tiab] | Small ethnic populations.tw.kf. | Small ethnic populations.ti,ab. | Small ethnic populations.ti,ab. | Small ethnic populations.ti,ab. | “Small ethnic populations” |
| (masyarakat[Other Term] OR adat)[Other Term] | (masyarakat or adat).tw.kf. | (masyarakat or adat).ti,ab. | (masyarakat or adat).ti,ab. | (masyarakat or adat).ti,ab. | “masyarakat” OR “adat” |
| Pribumi[tiab] | Pribumi.tw.kf. | Pribumi.ti,ab. | Pribumi.ti,ab. | Pribumi.ti,ab. | “Pribumi” |
| (Adibasi[Other Term] OR janajati[Other Term] OR Adivasi*[Other Term] OR adhivasi)[Other Term] | (Adibasi or janajati or Adivasi* or adhivasi).tw.kf. | (Adibasi or janajati or Adivasi* or adhivasi).ti,ab. | (Adibasi or janajati or Adivasi* or adhivasi).ti,ab. | (Adibasi or janajati or Adivasi* or adhivasi).ti,ab. | “Adibasi” OR “janajati” OR “Adivasi*” OR “adhivasi” |
| "hill tribe"[other term] OR “hill people”[other term] | (hill tribe or hill people).tw.kf. | (hill tribe or hill people).ti,ab. | (hill tribe or hill people).ti,ab. | (hill tribe or hill people).ti,ab. | “hill tribe” OR “hill people” |
| Saulteaux[tiab] | Saulteaux.tw.kf. | Saulteaux.ti,ab. | Saulteaux.ti,ab. | Saulteaux.ti,ab. | “Saulteaux” |
| Cree[tiab] | Cree.tw.kf. | Cree.ti,ab. | Cree.ti,ab. | Cree.ti,ab. | “Cree” |
| Dene[tiab] | Dene.tw.kf. | Dene.ti,ab. | Dene.ti,ab. | Dene.ti,ab. | “Dene” |
| Salish[[tiab] | Salish.tw.kf. | Salish.ti,ab. | Salish.ti,ab. | Salish.ti,ab. | “Salish” |
| Blackfoot[tiab] | Blackfoot.tw.kf. | Blackfoot.ti,ab. | Blackfoot.ti,ab. | Blackfoot.ti,ab. | “Blackfoot” |
| Algonquin[tiab] | Algonquin.tw.kf. | Algonquin.ti,ab. | Algonquin.ti,ab. | Algonquin.ti,ab. | “Algonquin” |
| Ojibway[tiab] | Ojibway.tw.kf. | Ojibway.ti,ab. | Ojibway.ti,ab. | Ojibway.ti,ab. | “Ojibway” |
| Innu[tiab] | Innu.tw.kf. | Innu.ti,ab. | Innu.ti,ab. | Innu.ti,ab. | “Innu” |
| Mikmaq[tiab] OR Micmac[tiab] | (Mikmaq or micmac).tw.kf. | (Mikmaq or micmac).ti,ab. | (Mikmaq or micmac).ti,ab. | (Mikmaq or micmac).ti,ab. | “Mikmaq” OR “Micmac” |
| Mohawk[tiab] | Mohawk.tw.kf. | Mohawk.ti,ab. | Mohawk.ti,ab. | Mohawk.ti,ab. | “Mohawk” |
| navaho*[tiab] OR navajo*[tiab] | (navaho*or navajo*).tw.kf. | (navaho*or navajo*).ti,ab. | (navaho*or navajo*).ti,ab. | (navaho*or navajo*).ti,ab. | “navaho*” OR “navajo*” |
| Cherokee[tiab] | Cherokee.tw.kf. | Cherokee.ti,ab. | Cherokee.ti,ab. | Cherokee.ti,ab. | “Cherokee” |
| Sioux[tiab] | Sioux.tw.kf. | Sioux.ti,ab. | Sioux.ti,ab. | Sioux.ti,ab. | “Sioux” |
| Chippewa[tiab] | Chippewa.tw.kf. | Chippewa.ti,ab. | Chippewa.ti,ab. | Chippewa.ti,ab. | “Chippewa” |
| Choctaw[tiab] | Choctaw.tw.kf. | Choctaw.ti,ab. | Choctaw.ti,ab. | Choctaw.ti,ab. | “Choctaw” |
| Apache[tiab] | Apache.tw.kf. | Apache.ti,ab. | Apache.ti,ab. | Apache.ti,ab. | “Apache” |
| Pueblo[tiab] | Pueblo.tw.kf. | Pueblo.ti,ab. | Pueblo.ti,ab. | Pueblo.ti,ab. | “Pueblo” |
| Iroquois[tiab] | Iroquois.tw.kf. | Iroquois.ti,ab. | Iroquois.ti,ab. | Iroquois.ti,ab. | “Iroquois” |
| Creek adj (people* OR communit* OR tribe* or person or woman or man or individual*)[tiab] | Creek adj (people* OR communit* OR tribe* or person or woman or man or individual*).tw.kf. | Creek adj (people* OR communit* OR tribe* or person or woman or man or individual*).ti,ab. | Creek adj (people* OR communit* OR tribe* or person or woman or man or individual*).ti,ab. | Creek adj (people* OR communit* OR tribe* or person or woman or man or individual*).ti,ab. | TX Creek adj (people* OR communit* OR tribe* or person or woman or man or individual*)” |
| Blackfeet[tiab] OR Blackfoot[tiab] | Blackfeet OR Blackfoot.tw.kf. | Blackfeet OR Blackfoot.ti,ab. | Blackfeet OR Blackfoot.ti,ab. | Blackfeet OR Blackfoot.ti,ab. | “Blackfeet" OR "Blackfoot” |
| Maya*[tiab] | Maya*.tw.kf. | Maya*.ti,ab. | Maya*.ti,ab. | Maya*.ti,ab. | “Maya*” |
| Zapotec*[tiab] | Zapotec*.tw.kf. | Zapotec*.ti,ab. | Zapotec*.ti,ab. | Zapotec*.ti,ab. | “Zapotec*” |
| Tzetal*[tiab] | Tzetal*.tw.kf. | Tzetal*.tw.kf. | Tzetal*.tw.kf. | Tzetal*.tw.kf. | “Tzetal*” |
| Otomí*[tiab] | Otomí*.tw.kf. | Otomí*.tw.kf. | Otomí*.tw.kf. | Otomí*.tw.kf. | “Otomí*” |
| Mazatec*[tiab] | Mazatec*.tw.kf. | Mazatec*.tw.kf. | Mazatec*.tw.kf. | Mazatec*.tw.kf. | “Mazatec*” |
| Totonac*[tiab] | Totonac*.tw.kf. | Totonac*.tw.kf. | Totonac*.tw.kf. | Totonac*.tw.kf. | “Totonac*” |
| Mazahua*[tiab] | Mazahua*.tw.kf. | Mazahua*.tw.kf. | Mazahua*.tw.kf. | Mazahua*.tw.kf. | “Mazahua*” |
| Huastec*[tiab] | Huastec*.tw.kf. | Huastec*.tw.kf. | Huastec*.tw.kf. | Huastec*.tw.kf. | “Huastec*” |
| Chole*[tiab] | Chole*.tw.kf. | Chole*.ti,ab. | Chole*.ti,ab. | Chole*.ti,ab. | “Chole*” |
| Guanahatabey[tiab] | Guanahatabey.tw.kf. | Guanahatabey. ti,ab. | Guanahatabey. ti,ab. | Guanahatabey. ti,ab. | “Guanahatabey” |
| Ciboney[tiab] | Ciboney.tw.kf. | Ciboney.ti,ab. | Ciboney.ti,ab. | Ciboney.ti,ab. | “Ciboney” |
| (Taino or yamaye or Arawak or lokono or Lucayan or arahuacos)[tiab] | (Taino or yamaye or Arawak or lokono or Lucayan or arahuacos).tw.kf. | (Taino or yamaye or Arawak or lokono or Lucayan or arahuacos). ti,ab. | (Taino or yamaye or Arawak or lokono or Lucayan or arahuacos). ti,ab. | (Taino or yamaye or Arawak or lokono or Lucayan or arahuacos). ti,ab. | (Taino or yamaye or Arawak or lokono or Lucayan or arahuacos) |
| (carib or kalina or karina or galibi or caribe or karinya or caribes or Teleuyu)[tiab] | (carib or kalina or karina or galibi or caribe or karinya or caribes or Téleuyu).tw.kf. | (carib or kalina or karina or galibi or caribe or karinya or caribes or Téleuyu). ti,ab. | (carib or kalina or karina or galibi or caribe or karinya or caribes or Téleuyu). ti,ab. | (carib or kalina or karina or galibi or caribe or karinya or caribes or Téleuyu). ti,ab. | “carib” OR “kalian” OR “karina” OR “galibi” OR “Caribe” OR “karinya” OR “caribes” OR “Téleuyu” |
| (mayan or ketchi or mopan or yucatec* or qeqchi)[tiab] | (mayan or ketchi or mopan or yucatec* or qeqchi).tw.kf. | (mayan or ketchi or mopan or yucatec* or qeqchi).ti,ab. | (mayan or ketchi or mopan or yucatec* or qeqchi).ti,ab. | (mayan or ketchi or mopan or yucatec* or qeqchi).ti,ab. | “mayan” OR “ketchi” OR “mopan” OR “yucatec*” OR “qeqchi” |
| Garifuna*[tiab] | Garifuna*.tw.kf. | Garifuna*.ti,ab. | Garifuna*.ti,ab. | Garifuna*.ti,ab. | “Garifuna*” |
| (Encomienda* or Cuaraor Tacarigua or Arouca or Arauca)[tiab] | (Encomienda* or Cuaraor Tacarigua or Arouca or Arauca).tw.kf. | (Encomienda* or Cuaraor Tacarigua or Arouca or Arauca).ti,ab. | (Encomienda* or Cuaraor Tacarigua or Arouca or Arauca).ti,ab. | (Encomienda* or Cuaraor Tacarigua or Arouca or Arauca).ti,ab. | “Encomienda*” OR “Cuaraor Tacarigua” OR “Arouca” OR “Arauca” |
| Aonikenk[tiab] | Aonikenk.tw.kf. | Aonikenk.ti,ab. | Aonikenk.ti,ab. | Aonikenk.ti,ab. | “Aonikenk” |
| Kolla[tiab] | Kolla.tw.kf. | Kolla.ti,ab. | Kolla.ti,ab. | Kolla.ti,ab. | “Kolla” |
| Qom[tiab] | Qom.tw.kf. | Qom.ti,ab. | Qom.ti,ab. | Qom.ti,ab. | “Qom” |
| Wichi[tiab] | Wichi.tw.kf. | Wichi.ti,ab. | Wichi.ti,ab. | Wichi.ti,ab. | “Wichi” |
| Diaguita[tiab] | Diaguita.tw.kf. | Diaguita.ti,ab. | Diaguita.ti,ab. | Diaguita.ti,ab. | “Diaguita” |
| Mocovi[tiab] | Mocovi.tw.kf. | Mocovi.ti,ab. | Mocovi.ti,ab. | Mocovi.ti,ab. | “Mocovi” |
| Huarpe[tiab] | Huarpe.tw.kf. | Huarpe.ti,ab. | Huarpe.ti,ab. | Huarpe.ti,ab. | “Huarpe” |
| Chiquitano[tiab] | Chiquitano.tw.kf. | Chiquitano.ti,ab. | Chiquitano.ti,ab. | Chiquitano.ti,ab. | “Chiquitano” |
| Mojeno[tiab] | Mojeno.tw.kf. | Mojeno.ti,ab. | Mojeno.ti,ab. | Mojeno.ti,ab. | “Mojeno” |
| afro-bolivian*[tiab] | afro-bolivian*.tw.kf. | afro-bolivian*.ti,ab. | afro-bolivian*.ti,ab. | (afro bolivia*).ti,ab. | “afro bolivia*” |
| Guarayo[tiab] | Guarayo.tw.kf. | Guarayo.ti,ab. | Guarayo.ti,ab. | Guarayo.ti,ab. | “Guarayo” |
| Movima[tiab] | Movima.tw.kf. | Movima.ti,ab. | Movima.ti,ab. | Movima.ti,ab. | “Movima” |
| Tacama[tiab] | Tacama.tw.kf. | Tacama.ti,ab. | Tacama.ti,ab. | Tacama.ti,ab. | “Tacama” |
| Itonama[tiab] | Itonama.tw.kf. | Itonama.ti,ab. | Itonama.ti,ab. | Itonama.ti,ab. | “Itonama” |
| Kaiwa[tiab] | Kaiwa.tw.kf. | Kaiwa.ti,ab. | Kaiwa.ti,ab. | Kaiwa.ti,ab. | “Kaiwa.ti,ab” |
| Guajajara[tiab] | Guajajara.tw.kf. | Guajajara.ti,ab. | Guajajara.ti,ab. | Guajajara.ti,ab. | “Guajajara” |
| Kaingang[tiab] | Kaingang.tw.kf. | Kaingang.ti,ab. | Kaingang.ti,ab. | Kaingang.ti,ab. | “Kaingang” |
| Terena[tiab] | Terena.tw.kf. | Terena.ti,ab. | Terena.ti,ab. | Terena.ti,ab. | “Terena” |
| Ticuna[tiab] | Ticuna.tw.kf. | Ticuna.ti,ab. | Ticuna.ti,ab. | Ticuna.ti,ab. | “Ticuna” |
| Yanomami[tiab] | Yanomami.tw.kf. | Yanomami.ti,ab. | Yanomami.ti,ab. | Yanomami.ti,ab. | “Yanomami” |
| Mapuche[tiab] | Mapuche.tw.kf. | Mapuche.ti,ab. | Mapuche.ti,ab. | Mapuche.ti,ab. | “Mapuche” |
| Lican antai[tiab] | Lican antai.tw.kf. | Lican antai.ti,ab. | Lican antai.ti,ab. | Lican antai.ti,ab. | “Lican antai” |
| colla[tiab] | colla.tw.kf. | colla.ti,ab. | colla.ti,ab. | colla.ti,ab. | “colla” |
| (Yaganes or yagan or Yamana)[tiab] | (Yaganes or yagan or Yamana).tw.kf. | (Yaganes or yagan or Yamana).ti,ab. | (Yaganes or yagan or Yamana).ti,ab. | (Yaganes or yagan or Yamana).ti,ab. | “Yaganes” OR “yagan” OR “Yamana” |
| Kawesgar[tiab] | Kawesgar.tw.kf. | Kawesgar.ti,ab. | Kawesgar.ti,ab. | Kawesgar.ti,ab. | “Kawesgar” |
| Rapanui[other term]or rapa nui[tiab] | (Rapanui or rapa nui).tw.kf. | (Rapanui or rapa nui).ti,ab. | (Rapanui or rapa nui).ti,ab. | (Rapanui or rapa nui).ti,ab. | “Rapanui” OR “rapa nui” |
| Diaguita[tiab] | Diaguita.tw.kf. | Diaguita.ti,ab. | Diaguita.ti,ab. | Diaguita.ti,ab. | “Diaguita” |
| Wayuu [tiab] | (Wayuu).tw.kf. | (Wayuu).ti,ab. | (Wayuu).ti,ab. | (Wayuu).ti,ab. | “Wayuu” |
| Nasa tribe*[tiab] | nasa adj (people* OR communit* OR tribe* or person or woman or man or individual*).tw.kf. | nasa adj (people* OR communit* OR tribe* or person or woman or man or individual*).ti,ab. | nasa adj (people* OR communit* OR tribe* or person or woman or man or individual*).ti,ab. | nasa adj (people* OR communit* OR tribe* or person or woman or man or individual*).ti,ab. | nasa adj (people* OR communit* OR tribe* OR person OR woman OR man OR individual*) |
| pastos[tiab] | pastos.tw.kf. | pastos.ti,ab. | pastos.ti,ab. | pastos.ti,ab. | “pastos” |
| chami[tiab] | chami.tw.kf. | chami.ti,ab. | chami.ti,ab. | chami.ti,ab. | “chami” |
| embera[tiab] | embera.tw.kf. | embera.ti,ab. | embera.ti,ab. | embera.ti,ab. | “embera” |
| sikuani[tiab] | sikuani.tw.kf. | sikuani.ti,ab. | sikuani.ti,ab. | sikuani.ti,ab. | “sikuani” |
| pijao[tiab] | pijao.tw.kf. | pijao.ti,ab. | pijao.ti,ab. | pijao.ti,ab. | “pijao” |
| katio[tiab] | katio.tw.kf. | katio.ti,ab. | katio.ti,ab. | katio.ti,ab. | “katio” |
| (Zenu)[tiab] | (Zenu).tw.kf. | (Zenu).ti,ab. | (Zenu).ti,ab. | (Zenu).ti,ab. | “Zenu” |
| Quichua[Text Word] OR kichwa[Text Word] OR quechua)[Text Word] | (Quichua or kichwa or quechua).tw.kf. | (Quichua or kichwa or quechua).ti,ab. | (Quichua or kichwa or quechua).ti,ab. | (Quichua or kichwa or quechua).ti,ab. | “Quichua” OR “kichwa” OR “quechua” |
| Shuar[tiab] | Shuar.tw.kf. | Shuar.ti,ab. | Shuar.ti,ab. | Shuar.ti,ab. | “Shuar” |
| Chachi[tiab] | Chachi.tw.kf. | Chachi.ti,ab. | Chachi.ti,ab. | Chachi.ti,ab. | “Chachi” |
| Achuar[tiab] | Achuar.tw.kf. | Achuar.ti,ab. | Achuar.ti,ab. | Achuar.ti,ab. | “Achuar” |
| (Awa)[tiab] | (Awa).tw.kf. | (Awa) .ti,ab. | (Awa) .ti,ab. | (Awa) .ti,ab. | “Awa” |
| TsaChila [tiab] | TsaChila.tw.kf. | TsaChila.ti,ab. | TsaChila.ti,ab. | TsaChila.ti,ab. | “TsaChila” |
| (Andoa or andwa) [tiab] | (Andoa or andwa).tw.kf. | (Andoa or andwa) .ti,ab. | (Andoa or andwa) .ti,ab. | (Andoa or andwa).ti,ab. | “Andoa” OR “andwa” |
| Huaorani[tiab] | Huaorani.tw.kf. | Huaorani.ti,ab. | Huaorani.ti,ab. | Huaorani.ti,ab. | “Huaorani” |
| Cofan[tiab] | Cofan.tw.kf. | Cofan.ti,ab. | Cofan.ti,ab. | Cofan.ti,ab. | “Cofan” |
| Arekuna[tiab] | Arekuna.tw.kf. | Arekuna.ti,ab. | Arekuna.ti,ab. | Arekuna.ti,ab. | “Arekuna” |
| Akawaio[tiab] | Akawaio.tw.kf. | Akawaio.ti,ab. | Akawaio.ti,ab. | Akawaio.ti,ab. | “Akawaio” |
| Patamona[tiab] | Patamona.tw.kf. | Patamona.ti,ab. | Patamona.ti,ab. | Patamona.ti,ab. | “Patamona” |
| Macushi[Text Word] OR makushi[Text Word] OR makusi[Text Word] | (Macushi or makushi or makusi).tw.kf. | (Macushi or makushi or makusi) .ti,ab. | (Macushi or makushi or makusi) .ti,ab. | (Macushi or makushi or makusi).ti,ab. | “Macushi” OR “makushi” OR “makusi” |
| Waiwai[tiab] | Waiwai.tw.kf. | Waiwai.ti,ab. | Waiwai.ti,ab. | Waiwai.ti,ab. | “Waiwai” |
| Wapishana[tiab] | Wapishana.tw.kf. | Wapishana.ti,ab. | Wapishana.ti,ab. | Wapishana.ti,ab. | “Wapishana” |
| warao[tiab] | (Warao or warau).tw.kf. | (Warao or warau).ti,ab. | (Warao or warau).ti,ab. | (Warao or warau).ti,ab. | “Warao” OR “warau” |
| (Guarani or Guaranior mbya or ava or occidental or nandeva)[tiab] | (Guarani or Guaranior mbya or ava or occidental or nandeva).tw.kf. | (Guarani or Guaranior mbya or ava or occidental or nandeva).ti,ab. | (Guarani or Guaranior mbya or ava or occidental or nandeva).ti,ab. | (Guarani or Guaranior mbya or ava or occidental or nandeva).ti,ab. | “Guarani” OR “Guaranior mbya” OR “ava” OR “occidental” OR “nandeva” |
| Enlhet norte[tiab] | Enlhet norte.tw.kf. | Enlhet norte.ti,ab. | Enlhet norte.ti,ab. | Enlhet norte.ti,ab. | “Enlhet norte” |
| Enxet sur[tiab] | Enxet sur.tw.kf. | Enxet sur.ti,ab. | Enxet sur.ti,ab. | Enxet sur.ti,ab. | “Enxet sur” |
| Angaite[tiab] | Angaite .tw.kf. | Angaite.ti,ab. | Angaite.ti,ab. | Angaite.ti,ab. | “Angaite” |
| Sanapana[tiab] | Sanapana.tw.kf. | Sanapana.ti,ab. | Sanapana.ti,ab. | Sanapana.ti,ab. | “Sanapana” |
| Ayoreo[tiab] | Ayoreo .tw.kf. | Ayoreo.ti,ab. | Ayoreo.ti,ab. | Ayoreo.ti,ab. | “Ayoreo” |
| Nivacle[tiab] | Nivacle.tw.kf. | Nivacle.ti,ab. | Nivacle.ti,ab. | Nivacle.ti,ab. | “Nivacle” |
| Toba maskoy[tiab] | Toba maskoy.tw.kf. | Toba maskoy.ti,ab. | Toba maskoy.ti,ab. | Toba maskoy.ti,ab. | “Toba maskoy” |
| Zamuco[tiab] | Zamuco.tw.kf. | Zamuco.ti,ab. | Zamuco.ti,ab. | Zamuco.ti,ab. | “Zamuco” |
| Pai Tavytera[text word] | (Pai-Tavytera).tw.kf. | (Pai-Tavytera).ti,ab. | (Pai-Tavytera).ti,ab. | Pai-Tavytera.ti,ab. | “Pai-Tavytera” |
| Aimara[Other Term] OR Aymara[Other Term] | (Aimara or Aymara) .tw.kf. | (Aimara or Aymara).ti,ab. | (Aimara or Aymara).ti,ab. | (Aimara or Aymara).ti,ab. | “Aimara” OR “Aymara” |
| Amazonia*[tiab] | Amazonia*.tw.kf. | Amazonia*.ti,ab. | Amazonia*.ti,ab. | Amazonia*.ti,ab. | “Amazonia*” |
| Ashaninka[Other Term] OR Asheninka[Other Term] | (Ashaninka or Asheninka) .tw.kf. | (Ashaninka or Asheninka).ti,ab. | (Ashaninka or Asheninka).ti,ab. | (Ashaninka or Asheninka).ti,ab. | “Ashaninka OR “Asheninka” |
| Awajun[tiab] | Awajun.tw.kf. | Awajun.ti,ab. | Awajun.ti,ab. | Awajun.ti,ab. | “Awajun” |
| Shipibo-Konibo[tiab] | Shipibo-Konibo.tw.kf. | Shipibo-Konibo.ti,ab. | Shipibo-Konibo.ti,ab. | Shipibo-Konibo.ti,ab. | “Shipibo-Konibo” |
| Tiriyo[Title/Abstract] OR trio[Title/Abstract] OR tirio[Title/Abstract] OR tareno[Title/Abstract] | (Tiriyo or trio or tirio or tareno).tw.kf. | (Tiriyo or trio or tirio or tareno).ti,ab. | (Tiriyo or trio or tirio or tareno).ti,ab. | (Tiriyo or trio or tirio or tareno).ti,ab. | “Tiriyo” OR “trio” OR “tirio” OR “tareno” |
| Wayana [tiab] | Wayana.tw.kf. | Wayana.ti,ab. | Wayana.ti,ab. | Wayana.ti,ab. | “Wayana” |
| Akurio [tiab] | Akurio.tw.kf. | Akurio.ti,ab. | Akurio.ti,ab. | Akurio.ti,ab. | “Akurio” |
| Guajiro [tiab] | Guajiro.tw.kf. | Guajiro.ti,ab. | Guajiro.ti,ab. | Guajiro.ti,ab. | “Guajiro” |
| Pemon [tiab] | Pemon.tw.kf. | Pemon.ti,ab. | Pemon.ti,ab. | Pemon.ti,ab. | “Pemon” |
| Jivi [tiab] | Jivi.tw.kf. | Jivi.ti,ab. | Jivi.ti,ab. | Jivi.ti,ab. | “Jivi” |
| Guajibo [tiab] | Guajibo.tw.kf. | Guajibo.ti,ab. | Guajibo.ti,ab. | Guajibo.ti,ab. | “Guajibo” |
| Kumanagoto [tiab] | Kumanagoto.tw.kf. | Kumanagoto.ti,ab. | Kumanagoto.ti,ab. | Kumanagoto.ti,ab. | “Kumanagoto” |
| Anu[tiab] | Anu.tw.kf. | Anu.ti,ab. | Anu.ti,ab. | Anu.ti,ab. | “Anu” |
| paraujona[tiab] | paraujona.tw.kf. | paraujona.ti,ab. | paraujona.ti,ab. | paraujona.ti,ab. | “paraujona” |
| Marrons[tiab] | Marrons.tw.kf. | Marrons.ti,ab. | Marrons.ti,ab. | Marrons.ti,ab. | “Marrons” |
| Bushinengue[tiab] | Bushinengue.tw.kf. | Bushinengue.ti,ab. | Bushinengue.ti,ab. | Bushinengue.ti,ab. | “Bushinengue” |
| Pahikweneh[tiab] | Pahikweneh.tw.kf. | Pahikweneh.ti,ab. | Pahikweneh.ti,ab. | Pahikweneh.ti,ab. | “Pahikweneh” |
| Wayana[tiab] | Wayana.tw.kf. | Wayana.ti,ab. | Wayana.ti,ab. | Wayana.ti,ab. | “Wayana” |
| Wayamp[tiab] | Wayamp.i.tw.kf. | Wayamp.ti,ab. | Wayamp.ti,ab. | Wayamp.ti,ab. | “Wayamp” |
| Teko[tiab] | Teko.tw.kf. | Teko.ti,ab. | Teko.ti,ab. | Teko.ti,ab. | “Teko” |
| Apalai[tiab] | Apalai.tw.kf. | Apalai.ti,ab. | Apalai.ti,ab. | Apalai.ti,ab. | “Apalai” |
| inupiaq[Title/Abstract] OR inupiat[Title/Abstract] | (inupiaq or inupiat).tw.kf. | (inupiaq or inupiat).ti,ab. | (inupiaq or inupiat).ti,ab. | (inupiaq or inupiat).ti,ab. | “inupiaq” OR “Inupiat” |
| yupik[tiab] | (yupik).tw.kf. | (yupik).ti,ab. | (yupik).ti,ab. | (yupik).ti,ab. | “yupik” |
| aleut*[tiab] | aleut*.tw.kf. | aleut*.ti,ab. | aleut*.ti,ab. | aleut*.ti,ab. | “aleut*” |
| Haida[tiab] | Haida.tw.kf. | Haida.ti,ab. | Haida.ti,ab. | Haida.ti,ab. | “Haida” |
| Tsimshian[Title/Abstract] OR Tlingit[Title/Abstract] | (Tsimshian or Tlingit).tw.kf. | (Tsimshian or Tlingit).ti,ab. | (Tsimshian or Tlingit).ti,ab. | (Tsimshian or Tlingit).ti,ab. | “Tsimshian” OR “Tlingit |
| samoyed*[tiab] | samoyed*.tw.kf. | samoyed*.ti,ab. | samoyed*.ti,ab. | samoyed*.ti,ab. | “samoyed*” |
| Ket[Title/Abstract] OR kenisei[Title/Abstract] | (Ket or kenisei).tw.kf. | (Ket or kenisei) .ti,ab. | (Ket or kenisei) .ti,ab. | (Ket or kenisei) .ti,ab. | “Ket” OR “kenisei” |
| Telengit[Title/Abstract] OR teleut[Title/Abstract] | (Telengit or teleut).tw.kf. | (Telengit or teleut) .ti,ab. | (Telengit or teleut) .ti,ab. | (Telengit or teleut) .ti,ab. | “Telengit” OR “teleut” |
| altai*[tiab] | altai*.tw.kf. | altai*.ti,ab. | altai*.ti,ab. | altai*.ti,ab. | “altai*” |
| Buryat[tiab] | Buryat.tw.kf. | Buryat.ti,ab. | Buryat.ti,ab. | Buryat.ti,ab. | “Buryat” |
| Yukaghir[Title/Abstract] OR yukagir[Title/Abstract] | (Yukaghir or yukagir).tw.kf. | (Yukaghir or yukagir).ti,ab. | (Yukaghir or yukagir).ti,ab. | (Yukaghir or yukagir).ti,ab. | “Yukaghir or yukagir” |
| Itelmen*[tiab] | Itelmen*.tw.kf. | Itelmen*.ti,ab. | Itelmen*.ti,ab. | Itelmen*.ti,ab. | “Itelmen*” |
| Alyutor*[tiab] | Alyutor*.tw.kf. | Alyutor*.ti,ab. | Alyutor*.ti,ab. | Alyutor*.ti,ab. | “Alyutor*” |
| evenk[Title/Abstract] | (Evenk or Even).tw.kf. | (Evenk or Even) .ti,ab. | (Evenk or Even) .ti,ab. | (Evenk or Even) .ti,ab. | “Evenk” |
| Chukchi[tiab] | Chukchi.tw.kf. | Chukchi.ti,ab. | Chukchi.ti,ab. | Chukchi.ti,ab. | “Chukchi” |
| Koryak*[tiab] | Koryak*.tw.kf. | Koryak*.ti,ab. | Koryak*.ti,ab. | Koryak*.ti,ab. | “Koryak*” |
| Nivkh[tiab] | Nivkh.tw.kf. | Nivkh.ti,ab. | Nivkh.ti,ab. | Nivkh.ti,ab. | “Nivkh” |
| Dusun[tiab] | Dusun.tw.kf. | Dusun.ti,ab. | Dusun.ti,ab. | Dusun.ti,ab. | “Dusun” |
| Belait[tiab] | Belait.tw.kf. | Belait.ti,ab. | Belait.ti,ab. | Belait.ti,ab. | “Belait” |
| Kedayan[tiab] | Kedayan.tw.kf. | Kedayan.ti,ab. | Kedayan.ti,ab. | Kedayan.ti,ab. | “Kedayan” |
| Murut[tiab] | Murut.tw.kf. | Murut.ti,ab. | Murut.ti,ab. | Murut.ti,ab. | “Murut” |
| Bisaya[Title/Abstract] OR bisayah[Title/Abstract] | (Bisaya.tw.kf. or bisayah).tw.kf. | (Bisaya.tw.kf. or bisayah).ti,ab. | (Bisaya.tw.kf. or bisayah).ti,ab. | (Bisaya.tw.kf. or bisayah).ti,ab. | “Bisaya” OR “bisayah” |
| Brunei[tiab] | Brunei.tw.kf. | Brunei.ti,ab. | Brunei.ti,ab. | Brunei.ti,ab. | “Brunei” |
| taingyinthar[tiab] | taingyinthar.tw.kf. | taingyinthar.ti,ab. | taingyinthar.ti,ab. | taingyinthar.ti,ab. | “taingyinthar” |
| "htanay taingyinthar" | Htanay taingyinthar.tw.kf. | Htanay taingyinthar.ti,ab. | Htanay taingyinthar.ti,ab. | Htanay taingyinthar.ti,ab. | “Htanay taingyinthar” |
| Rohingya*[tiab] | Rohingya*.tw.kf. | Rohingya*.ti,ab. | Rohingya*.ti,ab. | Rohingya*.ti,ab. | “Rohingya*” |
| Rakhine[tiab] | Rakhine.tw.kf. | Rakhine.ti,ab. | Rakhine.ti,ab. | Rakhine.ti,ab. | “Rakhine” |
| Karen[tiab] | Karen.tw.kf. | Karen.ti,ab. | Karen.ti,ab. | Karen.ti,ab. | “Karen” |
| Chin[tiab] | Chin.tw.kf. | Chin.ti,ab. | Chin.ti,ab. | Chin.ti,ab. | “Chin” |
| Naga[tiab] | Naga.tw.kf. | Naga.ti,ab. | Naga.ti,ab. | Naga.ti,ab. | “Naga” |
| Mon-Khmer[tiab] | (Mon-Khmer).tw.kf. | (Mon-Khmer).ti,ab. | (Mon-Khmer).ti,ab. | (Mon-Khmer).ti,ab. | “Mon-Khmer” |
| Chun chiet[tiab] | Chun chiet.tw.kf. | Chun chiet.ti,ab. | Chun chiet.ti,ab. | Chun chiet.ti,ab. | “Chun chiet” |
| Austronesien[tiab] | Austronésien.tw.kf. | Austronésien.ti,ab. | Austronésien.ti,ab. | Austronésien.ti,ab. | “Austronésien” |
| Jhabel[tiab] | Jhabel.tw.kf. | Jhabel.ti,ab. | Jhabel.ti,ab. | Jhabel.ti,ab. | “Jhabel” |
| Kihal[tiab] | Kihal.tw.kf. | Kihal.ti,ab. | Kihal.ti,ab. | Kihal.ti,ab. | “Kihal” |
| pakistan[Title/Abstract] OR pakistani*[Title/Abstract] | (More) adj3 (pakistan OR pakistani*).tw.kf. | (More) adj3 (pakistan OR pakistani*).ti,ab. | (More) adj3 (pakistan OR pakistani*).ti,ab. | (More) adj3 (pakistan OR pakistani*).ti,ab. | "pakistan" OR "pakistani*" |
| Kutana[tiab] | Kutana.tw.kf. | Kutana.ti,ab. | Kutana.ti,ab. | Kutana.ti,ab. | “Kutana” |
| Ode[Title/Abstract] OR rajpoot[Title/Abstract] | (Ode or rajpoot).tw.kf. | (Ode or rajpoot).ti,ab. | (Ode or rajpoot).ti,ab. | (Ode or rajpoot).ti,ab. | (“Ode” OR “rajpoot”) |
| Musali[tiab] | Musali.tw.kf. | Musali.ti,ab. | Musali.ti,ab. | Musali.ti,ab. | “Musali” |
| Baloch[tiab] | Baloch.tw.kf. | Baloch.ti,ab. | Baloch.ti,ab. | Baloch.ti,ab. | “Baloch” |
| (Tribe* or tribal) adj3 (sindh* or gilgit baltistan or chitral or pothohar) | (Tribe* or tribal) adj3 (sindh* or gilgit baltistan or chitral or pothohar).tw.kf. | (Tribe* or tribal) adj3 (sindh* or gilgit baltistan or chitral or pothohar).ti,ab. | (Tribe* or tribal) adj3 (sindh* or gilgit baltistan or chitral or pothohar).ti,ab. | (Tribe* or tribal) adj3 (sindh* or gilgit baltistan or chitral or pothohar).ti,ab. | (Tribe* or tribal) adj3 (sindh* or gilgit baltistan or chitral or pothohar) |
| yuanjumin[tiab] | yuanjumin.tw.kf. | yuanjumin.ti,ab. | yuanjumin.ti,ab. | yuanjumin.ti,ab. | “yuanjumin” |
| Punti[tiab] | Punti.tw.kf. | Punti.ti,ab. | Punti.ti,ab. | Punti.ti,ab. | “Punti” |
| Hakka[tiab] | Hakka.tw.kf. | Hakka.ti,ab. | Hakka.ti,ab. | Hakka.ti,ab. | “Hakka” |
| Minzu[tiab] | Minzu.tw.kf. | Minzu.ti,ab. | Minzu.ti,ab. | Minzu.ti,ab. | “Minzu” |
| Zhuang[tiab] | Zhuang.tw.kf. | Zhuang.ti,ab. | Zhuang.ti,ab. | Zhuang.ti,ab. | “Zhuang” |
| Manchu[tiab] | Manchu.tw.kf. | Manchu.ti,ab. | Manchu.ti,ab. | Manchu.ti,ab. | “Manchu” |
| Hui[tiab] | Hui.tw.kf. | Hui.ti,ab. | Hui.ti,ab. | Hui.ti,ab. | “Hui” |
| Miao[tiab] | Miao.tw.kf. | Miao.ti,ab. | Miao.ti,ab. | Miao.ti,ab. | “Miao” |
| Uygur[tiab] | Uygur.tw.kf. | Uygur.ti,ab. | Uygur.ti,ab. | Uygur.ti,ab. | “Uygur” |
| Bedouin[tiab] | Bedouin.tw.kf. | Bedouin.ti,ab. | Bedouin.ti,ab. | Bedouin.ti,ab. | “Bedouin” |
| Marsh people[tiab] or marsh dweller*[tiab] | Marsh people or marsh dweller*.tw.kf. | Marsh people or marsh dweller*.ti,ab. | Marsh people or marsh dweller*.ti,ab. | Marsh people OR marsh dweller*.ti,ab. | (“Marsh people” OR “marsh dweller”) |
| Yazidis[tiab] | Yazidis.tw.kf. | Yazidis.ti,ab. | Yazidis.ti,ab. | Yazidis.ti,ab. | “Yazidis” |
| Turkmen*[tiab] | Turkmen*.tw.kf. | Turkmen*.ti,ab. | Turkmen*.ti,ab. | Turkmen*.ti,ab. | “Turkmen*” |
| Talysh[tiab] | Talysh.tw.kf. | Talysh.ti,ab. | Talysh.ti,ab. | Talysh.ti,ab. | “Talysh” |
| Mazanderani [tiab] or tabari [tiab] | (Mazanderani or tabari).tw.kf. | (Mazanderani or tabari).ti,ab. | (Mazanderani or tabari).ti,ab. | (Mazanderani or tabari).ti,ab. | (“Mazanderani” OR “tabari”) |
| Mandaeans[tiab] | Mandaeans.tw.kf. | Mandaeans.ti,ab. | Mandaeans.ti,ab. | Mandaeans.ti,ab. | “Mandaeans” |
| Gilak*[tiab] | Gilak*.tw.kf. | Gilak*.ti,ab. | Gilak*.ti,ab. | Gilak*.ti,ab. | “Gilak*” |
| Copt*[tiab] | Copt*.tw.kf. | Copt*.ti,ab. | Copt*.ti,ab. | Copt*.ti,ab. | “Copt*” |
| Kyrgyz[tiab] or Kyrgyz[tiab] or Kirghiz[tiab] | (Kyrgyz or Kyrghyz or Kirghiz).tw.kf. | (Kyrgyz or Kyrghyz or Kirghiz).ti,ab. | (Kyrgyz or Kyrghyz or Kirghiz).ti,ab. | (Kyrgyz or Kyrghyz or Kirghiz).ti,ab. | “Kyrgyz”  OR “Kyrghyz” OR“Kirghiz” |
| Kurd*[tiab] | Kurd*.tw.kf. | Kurd*.ti,ab. | Kurd*.ti,ab. | Kurd*.ti,ab. | “Kurd*” |
| Assyrian*[tiab] | Assyrian*.tw.kf. | Assyrian*.ti,ab. | Assyrian*.ti,ab. | Assyrian*.ti,ab. | “Assyrian*” |
| Chaldean*[tiab] | Chaldean*.tw.kf. | Chaldean*.ti,ab. | Chaldean*.ti,ab. | Chaldean*.ti,ab. | “Chaldean*” |
| Chagossian*[tiab] | Chagossian*.tw.kf. | Chagossian*.ti,ab. | Chagossian*.ti,ab. | Chagossian*.ti,ab. | “Chagossian*” |
| Tetum[tiab] | Tetum.tw.kf. | Tetum.ti,ab. | Tetum.ti,ab. | Tetum.ti,ab. | “Tetum” |
| Mambai[tiab] | Mambai.tw.kf. | Mambai.ti,ab. | Mambai.ti,ab. | Mambai.ti,ab. | “Mambai” |
| Tukudede[tiab] | Tukudede.tw.kf. | Tukudede.ti,ab. | Tukudede.ti,ab. | Tukudede.ti,ab. | “Tukudede” |
| Galoli[tiab] | Galoli.tw.kf. | Galoli.ti,ab. | Galoli.ti,ab. | Galoli.ti,ab. | “Galoli” |
| Kenmak[tiab] | Kenmak.tw.kf. | Kenmak.ti,ab. | Kenmak.ti,ab. | Kenmak.ti,ab. | “Kenmak” |
| Baikeno[tiab] | Baikeno.tw.kf. | Baikeno.ti,ab. | Baikeno.ti,ab. | Baikeno.ti,ab. | “Baikeno” |
| Bhil[tiab] | Bhil.tw.kf. | Bhil.ti,ab. | Bhil.ti,ab. | Bhil.ti,ab. | “Bhil” |
| Gond[tiab] | Gond.tw.kf. | Gond.ti,ab. | Gond.ti,ab. | Gond.ti,ab. | “Gond” |
| Santal[tiab] | Santal.tw.kf. | Santal.ti,ab. | Santal.ti,ab. | Santal.ti,ab. | “Santal” |
| Mina[tiab] | Mina.tw.kf. | Mina.ti,ab. | Mina.ti,ab. | Mina.ti,ab. | “Mina” |
| Naikda[tiab] | Naikda.tw.kf. | Naikda.ti,ab. | Naikda.ti,ab. | Naikda.ti,ab. | “Naikda” |
| Oraon[tiab] | Oraon.tw.kf. | Oraon.ti,ab. | Oraon.ti,ab. | Oraon.ti,ab. | “Oraon” |
| Sugali*[tiab] | Sugali*.tw.kf. | Sugali*.ti,ab. | Sugali*.ti,ab. | Sugali*.ti,ab. | “Sugali*” |
| Munda[tiab] | Munda.tw.kf. | Munda.ti,ab. | Munda.ti,ab. | Munda.ti,ab. | “Munda” |
| Naga[tiab] | Naga.tw.kf. | Naga.ti,ab. | Naga.ti,ab. | Naga.ti,ab. | “Naga” |
| Khond[tiab] | Khond.tw.kf. | Khond.ti,ab. | Khond.ti,ab. | Khond.ti,ab. | “Khond” |
| Javanese[tiab] | Javanese.tw.kf. | Javanese.ti,ab. | Javanese.ti,ab. | Javanese.ti,ab. | “Javanese” |
| Sundanese[tiab] | Sundanese.tw.kf. | Sundanese.ti,ab. | Sundanese.ti,ab. | Sundanese.ti,ab. | “Sundanese” |
| Batak[tiab] | Batak.tw.kf. | Batak.ti,ab. | Batak.ti,ab. | Batak.ti,ab. | “Batak” |
| Madurese[tiab] | Madurese.tw.kf. | Madurese.ti,ab. | Madurese.ti,ab. | Madurese.ti,ab. | “Madurese” |
| vedda[tiab] or wanniyalaeto[tiab] | (vedda or wanniyalaeto).tw.kf. | (vedda or wanniyalaeto).ti,ab. | (vedda or wanniyalaeto).ti,ab. | (vedda or wanniyalaeto).ti,ab. | (“vedda” OR “wanniyalaeto”) |
| tamil[tiab] | tamil.tw.kf. | tamil.ti,ab. | tamil.ti,ab. | tamil.ti,ab. | “tamil” |
| moors[tiab] | moors.tw.kf. | moors.ti,ab. | moors.ti,ab. | moors.ti,ab. | “moors” |
| malay*[tiab] | malay*.tw.kf. | malay*.ti,ab. | malay*.ti,ab. | malay*.ti,ab. | “malay*” |
| burghers[tiab] | burghers.tw.kf. | burghers.ti,ab. | burghers.ti,ab. | burghers.ti,ab. | “burghers” |
| chetty[tiab] | chetty.tw.kf. | chetty.ti,ab. | chetty.ti,ab. | chetty.ti,ab. | “chetty” |
| bharatha[tiab] | bharatha.tw.kf. | bharatha.ti,ab. | bharatha.ti,ab. | bharatha.ti,ab. | “bharatha” |
| Brahmins[tiab] | Brahmins.tw.kf. | Brahmins.ti,ab. | Brahmins.ti,ab. | Brahmins.ti,ab. | “Brahmins” |
| Chhetris[tiab] | Chhetris.tw.kf. | Chhetris.ti,ab. | Chhetris.ti,ab. | Chhetris.ti,ab. | “Chhetris” |
| Terai[tiab] | Terai.tw.kf. | Terai.ti,ab. | Terai.ti,ab. | Terai.ti,ab. | “Terai” |
| Dalits[tiab] | Dalits.tw.kf. | Dalits.ti,ab. | Dalits.ti,ab. | Dalits.ti,ab. | “Dalits” |
| Newars[tiab] | Newars.tw.kf. | Newars.ti,ab. | Newars.ti,ab. | Newars.ti,ab. | “Newars” |
| Magar[tiab] | Magar.tw.kf. | Magar.ti,ab. | Magar.ti,ab. | Magar.ti,ab. | “Magar” |
| Tamang[tiab] | Tamang.tw.kf. | Tamang.ti,ab. | Tamang.ti,ab. | Tamang.ti,ab. | “Tamang” |
| Kumal[tiab] | Kumal.tw.kf. | Kumal.ti,ab. | Kumal.ti,ab. | Kumal.ti,ab. | “Kumal” |
| Sunuwar[tiab] | Sunuwar.tw.kf. | Sunuwar.ti,ab. | Sunuwar.ti,ab. | Sunuwar.ti,ab. | “Sunuwar” |
| Tharu[tiab] | Tharu.tw.kf. | Tharu.ti,ab. | Tharu.ti,ab. | Tharu.ti,ab. | “Tharu” |
| Igorot[tiab] | Igorot.tw.kf. | Igorot.ti,ab. | Igorot.ti,ab. | Igorot.ti,ab. | “Igorot” |
| Lumad[tiab] | Lumad.tw.kf. | Lumad.ti,ab. | Lumad.ti,ab. | Lumad.ti,ab. | “Lumad” |
| Mangyan[tiab] | Mangyan.tw.kf. | Mangyan.ti,ab. | Mangyan.ti,ab. | Mangyan.ti,ab. | “Mangyan” |
| Pingpu[tiab] | Pingpu.tw.kf. | Pingpu.ti,ab. | Pingpu.ti,ab. | Pingpu.ti,ab. | “Pingpu” |
| Amis[tiab] | Amis.tw.kf. | Amis.ti,ab. | Amis.ti,ab. | Amis.ti,ab. | “Amis” |
| Atayal[tiab] | Atayal.tw.kf. | Atayal.ti,ab. | Atayal.ti,ab. | Atayal.ti,ab. | “Atayal” |
| Paiwan[tiab] | Paiwan.tw.kf. | Paiwan.ti,ab. | Paiwan.ti,ab. | Paiwan.ti,ab. | “Paiwan” |
| Bunun[tiab] | Bunun.tw.kf. | Bunun.ti,ab. | Bunun.ti,ab. | Bunun.ti,ab. | “Bunun” |
| Pinuyumayan[tiab] | Pinuyumayan.tw.kf. | Pinuyumayan.ti,ab. | Pinuyumayan.ti,ab. | Pinuyumayan.ti,ab. | “Pinuyumayan” |
| Rukai[tiab] | Rukai.tw.kf. | Rukai.ti,ab. | Rukai.ti,ab. | Rukai.ti,ab. | “Rukai” |
| Tsou[tiab] | Tsou.tw.kf. | Tsou.ti,ab. | Tsou.ti,ab. | Tsou.ti,ab. | “Tsou” |
| Saisiyat[tiab] | Saisiyat.tw.kf. | Saisiyat.ti,ab. | Saisiyat.ti,ab. | Saisiyat.ti,ab. | “Saisiyat” |
| Truku[tiab] | Truku.tw.kf. | Truku.ti,ab. | Truku.ti,ab. | Truku.ti,ab. | “Truku” |
| Sediq[tiab] | Sediq.tw.kf. | Sediq.ti,ab. | Sediq.ti,ab. | Sediq.ti,ab. | “Sediq” |
| Karen[tiab] | Karen.tw.kf. | Karen.ti,ab. | Karen.ti,ab. | Karen.ti,ab. | “Karen” |
| Hmong[tiab] | Hmong.tw.kf. | Hmong.ti,ab. | Hmong.ti,ab. | Hmong.ti,ab. | “Hmong” |
| Lahu[tiab] | Lahu.tw.kf. | Lahu.ti,ab. | Lahu.ti,ab. | Lahu.ti,ab. | “Lahu” |
| Akha[tiab] | Akha.tw.kf. | Akha.ti,ab. | Akha.ti,ab. | Akha.ti,ab. | “Akha” |
| Yao[tiab] | Yao.tw.kf. | Yao.ti,ab. | Yao.ti,ab. | Yao.ti,ab. | “Yao” |
| Htin[tiab] | Htin.tw.kf. | Htin.ti,ab. | Htin.ti,ab. | Htin.ti,ab. | “Htin” |
| Ainu[tiab] or aynu[tiab] | (Ainu or aynu).tw.kf. | (Ainu or aynu).ti,ab. | (Ainu or aynu).ti,ab. | (Ainu or aynu).ti,ab. | (“Ainu” OR “aynu”) |
| Okinawan*[tiab] | Okinawan*.tw.kf. | Okinawan*.ti,ab. | Okinawan*.ti,ab. | Okinawan*.ti,ab. | “Okinawan*” |
| Orang Asal[tiab] or Orang Asli[tiab] | (Orang Asal or Orang Asli).tw.kf. | (Orang Asal or Orang Asli).ti,ab. | (Orang Asal or Orang Asli).ti,ab. | (Orang Asal or Orang Asli).ti,ab. | (“Orang Asal” OR “Orang Asli”) |
| Negritos[tiab] | Negritos.tw.kf. | Negritos.ti,ab. | Negritos.ti,ab. | Negritos.ti,ab. | “Negritos” |
| Senoi[tiab] | Senoi.tw.kf. | Senoi.ti,ab. | Senoi.ti,ab. | Senoi.ti,ab. | “Senoi” |
| Proto-Malays[tiab] | Proto-Malays.tw.kf. | Proto-Malays.ti,ab. | Proto-Malays.ti,ab. | Proto-Malays.ti,ab. | “Proto-Malays” |
| Dayaks[tiab] | Dayaks.tw.kf. | Dayaks.ti,ab. | Dayaks.ti,ab. | Dayaks.ti,ab. | “Dayaks” |
| Iban[tiab] | Iban.tw.kf. | Iban.ti,ab. | Iban.ti,ab. | Iban.ti,ab. | “Iban” |
| Bidayuh[tiab] | Bidayuh.tw.kf. | Bidayuh.ti,ab. | Bidayuh.ti,ab. | Bidayuh.ti,ab. | “Bidayuh” |
| Kadazan-Dusun[tiab] | Kadazan-Dusun.tw.kf. | Kadazan-Dusun.ti,ab. | Kadazan-Dusun.ti,ab. | Kadazan-Dusun.ti,ab. | “Kadazan-Dusun” |
| Bajau[tiab] | Bajau.tw.kf. | Bajau.ti,ab. | Bajau.ti,ab. | Bajau.ti,ab. | “Bajau” |
| Murut[tiab] | Murut.tw.kf. | Murut.ti,ab. | Murut.ti,ab. | Murut.ti,ab. | “Murut” |
| Brokpa[tiab] | Brokpa.tw.kf. | Brokpa.ti,ab. | Brokpa.ti,ab. | Brokpa.ti,ab. | “Brokpa” |
| Lepcha[tiab] | Lepcha.tw.kf. | Lepcha.ti,ab. | Lepcha.ti,ab. | Lepcha.ti,ab. | “Lepcha” |
| Doya[tiab] | Doya.tw.kf. | Doya.ti,ab. | Doya.ti,ab. | Doya.ti,ab. | “Doya” |
| Kheng[tiab] | Kheng.tw.kf. | Kheng.ti,ab. | Kheng.ti,ab. | Kheng.ti,ab. | “Kheng” |
| Tibet*[tiab] | Tibet*.tw.kf. | Tibet*.ti,ab. | Tibet*.ti,ab. | Tibet*.ti,ab. | “Tibet*” |
| Toktop[tiab] | Toktop.tw.kf. | Toktop.ti,ab. | Toktop.ti,ab. | Toktop.ti,ab. | “Toktop” |
| Maasai[tiab] | Maasai.tw.kf. | Maasai.ti,ab. | Maasai.ti,ab. | Maasai.ti,ab. | “Maasai” |
| Borana[tiab] | Borana.tw.kf. | Borana.ti,ab. | Borana.ti,ab. | Borana.ti,ab. | “Borana” |
| Gabra[tiab] | Gabra.tw.kf. | Gabra.ti,ab. | Gabra.ti,ab. | Gabra.ti,ab. | “Gabra” |
| Samburu[tiab] | Samburu.tw.kf. | Samburu.ti,ab. | Samburu.ti,ab. | Samburu.ti,ab. | “Samburu” |
| Turkana[tiab] | Turkana.tw.kf. | Turkana.ti,ab. | Turkana.ti,ab. | Turkana.ti,ab. | “Turkana” |
| Pokot[tiab] | Pokot.tw.kf. | Pokot.ti,ab. | Pokot.ti,ab. | Pokot.ti,ab. | “Pokot” |
| Karamajong[tiab] | Karamajong.tw.kf. | Karamajong.ti,ab. | Karamajong.ti,ab. | Karamajong.ti,ab. | “Karamajong” |
| Batwa[tiab] | Batwa.tw.kf. | Batwa.ti,ab. | Batwa.ti,ab. | Batwa.ti,ab. | “Batwa” |
| Rendille[tiab] | Rendille.tw.kf. | Rendille.ti,ab. | Rendille.ti,ab. | Rendille.ti,ab. | “Rendille” |
| Ogiek[tiab] | Ogiek.tw.kf. | Ogiek.ti,ab. | Ogiek.ti,ab. | Ogiek.ti,ab. | “Ogiek” |
| Amazigh[tiab] | Amazigh.tw.kf. | Amazigh.ti,ab. | Amazigh.ti,ab. | Amazigh.ti,ab. | “Amazigh” |
| Imazighn[tiab] | Imazighn.tw.kf. | Imazighn.ti,ab. | Imazighn.ti,ab. | Imazighn.ti,ab. | “Imazighn” |
| Berber[tiab] | Berber.tw.kf. | Berber.ti,ab. | Berber.ti,ab. | Berber.ti,ab. | “Berber” |
| San[tiab] | San.tw.kf. | San.ti,ab. | San.ti,ab. | San.ti,ab. | “San” |
| Khoekhoe[tiab] | Khoekhoe.tw.kf. | Khoekhoe.ti,ab. | Khoekhoe.ti,ab. | Khoekhoe.ti,ab. | “Khoekhoe” |
| Mbororo[tiab] | Mbororo.tw.kf. | Mbororo.ti,ab. | Mbororo.ti,ab. | Mbororo.ti,ab. | “Mbororo” |
| Bororo[tiab] | Bororo.tw.kf. | Bororo.ti,ab. | Bororo.ti,ab. | Bororo.ti,ab. | “Bororo” |
| Wodaabe[tiab] | Wodaabe.tw.kf. | Wodaabe.ti,ab. | Wodaabe.ti,ab. | Wodaabe.ti,ab. | “Wodaabe” |
| Tuareg[tiab] | Tuareg.tw.kf. | Tuareg.ti,ab. | Tuareg.ti,ab. | Tuareg.ti,ab. | “Tuareg” |
| Tubu[tiab] | Tubu.tw.kf. | Tubu.ti,ab. | Tubu.ti,ab. | Tubu.ti,ab. | “Tubu” |
| Teda[tiab] | Teda.tw.kf. | Teda.ti,ab. | Teda.ti,ab. | Teda.ti,ab. | “Teda” |
| Daza[tiab] | Daza.tw.kf. | Daza.ti,ab. | Daza.ti,ab. | Daza.ti,ab. | “Daza” |
| pygmies[tiab] | pygmies.tw.kf. | pygmies.ti,ab. | pygmies.ti,ab. | pygmies.ti,ab. | “pygmies” |
| Aka[tiab] | Aka.tw.kf. | Aka.ti,ab. | Aka.ti,ab. | Aka.ti,ab. | “Aka” |
| Bagyeli[tiab] | Bagyeli.tw.kf. | Bagyeli.ti,ab. | Bagyeli.ti,ab. | Bagyeli.ti,ab. | “Bagyeli” |
| Bakola[tiab] | Bakola.tw.kf. | Bakola.ti,ab. | Bakola.ti,ab. | Bakola.ti,ab. | “Bakola” |
| Bakoya[tiab] | Bakoya.tw.kf. | Bakoya.ti,ab. | Bakoya.ti,ab. | Bakoya.ti,ab. | “Bakoya” |
| Baka[tiab] | Baka.tw.kf. | Baka.ti,ab. | Baka.ti,ab. | Baka.ti,ab. | “Baka” |
| Babenjelle[tiab] | Babenjelle.tw.kf. | Babenjelle.ti,ab. | Babenjelle.ti,ab. | Babenjelle.ti,ab. | “Babenjelle” |
| Babi[tiab] | Babi.tw.kf. | Babi.ti,ab. | Babi.ti,ab. | Babi.ti,ab. | “Babi” |
| Bacwa[tiab] | Bacwa.tw.kf. | Bacwa.ti,ab. | Bacwa.ti,ab. | Bacwa.ti,ab. | “Bacwa” |
| Babongo[tiab] | Babongo.tw.kf. | Babongo.ti,ab. | Babongo.ti,ab. | Babongo.ti,ab. | “Babongo” |
| Bambuti[tiab] | Bambuti.tw.kf. | Bambuti.ti,ab. | Bambuti.ti,ab. | Bambuti.ti,ab. | “Bambuti” |
| bushmen[tiab] | bushmen.tw.kf. | bushmen.ti,ab. | bushmen.ti,ab. | bushmen.ti,ab. | “bushmen” |
| (Torres strait island*)[tiab] | (Torres strait island*).ti,ab,kf. | (Torres strait island*).ti,ab. | (Torres strait island*).ti,ab. | (Torres strait island*).ti,ab. | “Torres strait island*” |
| murray island*[tiab] or mer island*[tiab] | (murray island* or mer island*).tw.kf. | (murray island* or mer island*).ti,ab. | (murray island* or mer island*).ti,ab. | (murray island* or mer island*).ti,ab. | “murray island*” OR “mer island*” |
| native Hawaii*[tiab] or kanaka maoli[tiab] | (native Hawaii* or kanaka maoli).tw.kf. | (native Hawaii* or kanaka maoli).ti,ab. | (native Hawaii* or kanaka maoli).ti,ab. | (native Hawaii* or kanaka maoli).ti,ab. | “native Hawaii*” OR “kanaka maoli” |
| Kanak*[tiab] | Kanak*.tw.kf. | Kanak*.ti,ab. | Kanak*.ti,ab. | Kanak*.ti,ab. | “Kanak*” |
| Chamorro*[tiab] | Chamorro*.tw.kf. | Chamorro*.ti,ab. | Chamorro*.ti,ab. | Chamorro*.ti,ab. | “Chamorro*” |
| maori*[tiab] | (maori*).tw.kf. | (maori*).ti,ab. | (maori*).ti,ab. | (maori*).ti,ab. | “maori*” |
| Moriori[tiab] | Moriori.tw.kf. | Moriori.ti,ab. | Moriori.ti,ab. | Moriori.ti,ab. | “Moriori” |
| Niue*[tiab] | Niue*.tw.kf. | Niue*.ti,ab. | Niue*.ti,ab. | Niue*.ti,ab. | “Niue*” |
| Rapanui[tiab] | Rapanui.tw.kf. | Rapanui.ti,ab. | Rapanui.ti,ab. | Rapanui.ti,ab. | “Rapanui” |
| Rotuma*[tiab] | Rotuma*.tw.kf. | Rotuma*.ti,ab. | Rotuma*.ti,ab. | Rotuma*.ti,ab. | “Rotuma*” |
| samoa*[tiab] | samoa*.tw.kf. | samoa*.ti,ab. | samoa*.ti,ab. | samoa*.ti,ab. | “samoa*” |
| Tuvalu*[tiab] | Tuvalu*.tw.kf. | Tuvalu*.ti,ab. | Tuvalu*.ti,ab. | Tuvalu*.ti,ab. | “Tuvalu*” |
| Marshall*[tiab] | Marshall*.tw.kf. | Marshall*.ti,ab. | Marshall*.ti,ab. | Marshall*.ti,ab. | “Marshall*” |
| Tahiti*[tiab] | Tahiti*.tw.kf. | Tahiti*.ti,ab. | Tahiti*.ti,ab. | Tahiti*.ti,ab. | “Tahiti*” |
| tonga*[tiab] | tonga*.tw.kf. | tonga*.ti,ab. | tonga*.ti,ab. | tonga*.ti,ab. | “tonga*” |
| Melanesia*[tiab] | Melanesia*.tw.kf. | Melanesia*.ti,ab. | Melanesia*.ti,ab. | Melanesia*.ti,ab. | “Melanesia*” |
| Micronesia*[tiab] | Micronesia*.tw.kf. | Micronesia*.ti,ab. | Micronesia*.ti,ab. | Micronesia*.ti,ab. | “Micronesia*” |
| Papua*[tiab] | Papua*.tw.kf. | Papua*.ti,ab. | Papua*.ti,ab. | Papua*.ti,ab. | “Papua*” |
| tiwi[tiab] | tiwi.tw.kf. | tiwi.ti,ab. | tiwi.ti,ab. | tiwi.ti,ab. | “tiwi” |
| Scale Validation and Adaptation Terms | | | | | |
| “Validation” [MeSH] | Validation Study/ | Exp Test validity/ | Exp Validity/ | Methodology.sh. | MH "Interrater Reliability/MT") |
| “Reliability and validity” [MeSH] | Reliability.ti,ab | Exp Test reliability/ | Exp Reliability/ |  |  |
| “Reproducibility of results” [MeSH] | Reproducibility of results/ | Reproducibility*.ti,ab | Exp Reproducibility |  |  |
| "Cross cultural adaptation"[Other Term] | Cross cultural adaptation.mp. | Exp Cross cultural test adaptation/ | Adaptation/ | Adaptation/ | "Cultural adaptation" |
| "Idioms of distress"[Other Term] | Idioms of distress.mp. | Exp Figurative Language/ | Idioms of distress.mp. | Idioms of distress.mp. | “Idioms of distress” |
| “Cross cultural comparison*” [MeSH] | Exp Cross-cultural comparison/ | Exp Cross cultural differences/ | Cross cultural comparison*.ti,ab | Cross cultural differences/ | “Cross cultural comparison” |
| "Cultural sensitivity"[Other Term] | Cultural sensitivity.mp. | Exp Cultural sensitivity/ | Cultural sensitivity/ | Cultural sensitivity*.ti,ab | MH “Cultural sensitivity” |
| “Sensitivity and Specificity” [MeSH] | Sensitivity and Specificity/ | Exp Test sensitivity/ | “Sensitivity and specificity”/ | (Sensitivity and specificity).mp. | MH “Sensitivity and specificity” |
| “Predictive Value of Tests” [MeSH] | Predictive value of tests/ | Exp Predictive validity/ | Predictive validity/ | Predictive value.mp. | MH “Predictive value of tests” |
| “Translations” [MeSH] | Exp Translations/ | Exp Foreign language translation/ | Translation.mp. | Translation/ | MH “Translations” |
| “Language” [MeSH] | Language/ | Exp Native language/ | Language/ | Language/ | MH “Language/EP” |
| “Culturally Competent Care*” [MeSH] | Exp Culturally competent care/ | Cultural competence.mp. | Cultural competence/ | Cultural competence*.ti,ab | MH “Cultural competence” |
| “Cultural Competency/Psychology*”[MeSH] | Exp Cultural competency/px |  |  |  |  |
| “Patient Acceptance of Health Care/psychology” [MeSH] | “Patient acceptance of health care”/ | Patient acceptance.mp. | Patient acceptance.mp. | Patient acceptance.mp. | “Patient acceptance of health care” |
| “Focus groups/methods*” [MeSH] | Exp Focus groups/mt | Exp Focus Group/ | Focus group*.ti,ab | Focus group*.ti,ab | MH “Focus groups” |
| “Measure, outcome” [MeSH] | Measure*.ti,ab | Exp Measurement/ | Measurement/ | Measurement/ | “Measurement” |
| “Psychiatric Status Rating Scales*” [MeSH] | Exp Psychiatric status rating scales/ | Exp Rating scales/ | Rating scale/ | Rating scale.mp. | MH “Self-rating depression scale” |
| “Psychometrics” [MeSH] | Psychometrics/ | Exp Psychometrics/ | Psychometry/ | Psychometric*.mp. | MH “Psychometrics” |
| “Surveys and Questionnaires*”[MeSH] | Exp Surveys and Questionnaires/ | Exp Questionnaires/ | Questionnaire/ | Questionnaires.sh. | MH “Questionnaires” |
| [Depression Terms] combined with OR | | | | | |
| [Indigenous Terms] combined with OR | | | | | |
| [Scale Validation and Adaptation Terms] combined with OR | | | | | |
| [[Depression Terms] combined with OR] AND [[Indigenous Terms] combined with OR] AND [[Scale Validation and Adaptation Terms] combined with OR] | | | | | |
