# Supplementary material for: Safe and valid? A systematic review of the psychometric properties of culturally adapted depression scales for use among Indigenous populations
Source: Glob Ment Health (Camb). 2023 Sep 14;10:e60. doi: 10.1017/gmh.2023.52 (PMC10579654; doi:10.1017/gmh.2023.52)
Supplement: Yang et al. supplementary material 2 — Yang et al. supplementary material [file S2054425123000523sup002.docx]

**Supplemental 2**

*Quality criteria checklist for psychometric properties of depression scales*

| **Domain** | **Property** | **Definition of the Property** | **Measures/tests** | **Levels of Evidence Cut-offs** | | |
| --- | --- | --- | --- | --- | --- | --- |
|  |  |  |  | **Weak** | **Acceptable** | **Strong** |
| Characteristics of the adapted scale | Internal consistency reliability^1,2^ | Degree of the interrelatedness among the items | Item-item correlation, Item-total correlation, Cronbach’s alpha (α),  Intraclass correlation coefficient (ICC) | r <0.40; α<0.60; ICC < 0.40 | r = 0.40-0.70; α= 0.60-0.80; ICC= 0.40-0.75 | r > 0.70  α>0.80; ICC> 0.75 |
|  | Inter-Rater reliability^2,9,15^ | Degree to which different people administering the analysis yield the same result | Intraclass correlation coefficient (ICC), Pearson’s correlation (r) | r <0.40; ICC< 0.40 | r = 0.40-0.70; ICC = 0.40-0.75 | r> 0.70; ICC> 0.75 |
|  | Test-Retest reliability^1,2,^ | The degree to which assessments capture the construct to be measured if measured repeatedly | Dependent samples t-test; Intraclass correlation coefficient (ICC), Pearson’s correlation (r) | r <0.40; ICC< 0.40 | r = 0.40-0.70; ICC = 0.40-0.75 | r> 0.70; ICC> 0.75 |
| Criterion validity^13^ | Concurrent validity^1^ | Degree to which item scores presently correlate with measure that have previously been validated | Pearson correlation (r), p-value, Cohen’s κ | \| r \| <0.40; p-value≥ 0.05; κ<0.2 | \| r \| = 0.40-0.70; κ=0.2-0.6 | \| r \| > 0.70; p-value <0.05; κ>0.6-0.8 |
|  | Predictive validity^1^ | Degree to which item scores correlate with criterion at a future time | Pearson correlation (r), Point-biserial correlation; Beta coefficient | \| r \| <0.40; p-value ≥ 0.05; κ<0.2 | \| r \| = 0.40-0.70; κ=0.2-0.6 | \| r \| > 0.70; p-value <0.05; κ>0.6-0.8 |
| Comparison to the original scale | Cross-cultural validity^3,4,5,6^ | Degree of generalizability across settings/populations**-** demonstrated by the measurement invariance | Multi-group Confirmatory factor analysis (CFA), measurement invariance analysis | Several (>2) fit indices are below recommended in strong; p > 0.05) | Few (≤2) fit indices are below recommended in strong; CFI/ TLI < 0.95; WRMR 0.70-0.90; RMSEA= 0.05-0.08; SRMR= 0.08-0.10 | p-value <0.05  Fit indices:  CFI/ TLI > 0.95; RMSEA <0.05; SRMR <0.08; WRMR > 0.90; |
| Construct validity^16^ | Convergent validity^1,7,11,12, 13, 14^ | Degree to which a test measures the same attributes as other measures that purport to measure the same construct | Pearson correlation (r), Factor analysis (CFA and Factor loadings (FL)) | \| r \| <0.40; Several (>2) fit indices are below recommended in strong; p-value≥ 0.05 | \| r \| = 0.40-0.70; Few (≤2) fit indices are below recommended in strong | \| r \| > 0.70 Eigenvalue (EVA) ≥1.0; r(FL) ≥ 0.4; p-value <0.05  Fit indices:  RMSEA≤0.08; CFI/TLI>0.90; SRMR ≤ 0.10; WRMR > 0.90 |
|  | Incremental validity^8^ | Degree to which a new measure improves predictions over other existing methods of assessments | F or t-test; Variance Inflation Factor (VIF), Pearson’s correlation (r) | R^2^ increases (p > 0.05) |  | R^2^ increases (p < 0.05); VIF < 5 |
|  | Discriminant validity^1^ | Degree to which a test measures something different from what other available tests measure | Pearson’s correlation (r), p-value | r <0.40; p-value≥ 0.05 | r = 0.40-0.70 | r > 0.70, p-value<0.05 |
| Clinical utility^17,18^ | Sensitivity | Proportion of actual positives that are correctly identified as such | True positive detection rate | <70% | 70%-85% | > 85% |
|  | Specificity | Proportion of actual negatives that are correctly identified as such | True negative detection rate | <70% | 70%-85% | > 85% |
|  | Discrimination | The accuracy of a test to distinguish between diagnostic groups | Area under the Receiving Operating Curve (AUC) | AUC=0.40-0.70 | AUC=0.70-0.90 | AUC>0.90 |
| Diagnostic accuracy^17,19^ | Positive predictive value (PPV) | The proportions of true positive responses when the illness is present | Probability | <60% | 60%-85% | >85% |
|  | Negative predictive value (NPV) | The proportion of false negative responses when the illness is absent | Probability | <60% | 60%-85% | >85% |
|  | Likelihood Ratio (LR)^10^ | The ratio between the probability that the given test result is expected in an affected individual and the probability that the same result will occur in a healthy subject | Likelihood | LR= 1.0-3.0 | LR= 3.0-8.0 | LR> 8.0 |

**References**

1. Awang, Z. *A Handbook on SEM 2nd Edition Chapter 3*.
2. Cheung, G., & Rensvold, R. (2002). Evaluating Goodness-of-Fit Indexes for Testing Measurement Invariance. *Structural Equation Modeling*, *9*, 233-255. <https://doi.org/10.1207/S15328007SEM0902_5>
3. Craney, T. A., & Surles, J. G. (2002). Model-Dependent Variance Inflation Factor Cutoff Values. *Quality Engineering*, *14*(3), 391-403. <https://doi.org/10.1081/QEN-120001878>
4. Enderlein, G. (1988). Fleiss, J. L.: The Design and Analysis of Clinical Experiments. Wiley, New York – Chichester – Brislane – Toronto – Singapore 1986, 432 S., £38.35. *Biometrical Journal*, *30*(3), 304-304. <https://doi.org/10.1002/bimj.4710300308>
5. Han, Y., Zhang, J., Jiang, Z., & Shi, D. (2022). Is the Area Under Curve Appropriate for Evaluating the Fit of Psychometric Models? *Educational and Psychological Measurement*, 00131644221098182. <https://doi.org/10.1177/00131644221098182>
6. Hu L, B. P. (1999). Cutoff criteria for fit indices in covariance structure analysis: conventional criteria versus new alternatives. *Struct Equ Modeling.*, *6*, 1-55.
7. Jaeschke, R., Guyatt, G. H., & Sackett, D. L. (1994). Users' guides to the medical literature. III. How to use an article about a diagnostic test. B. What are the results and will they help me in caring for my patients? The Evidence-Based Medicine Working Group. *JAMA*, *271*(9), 703-707. <https://doi.org/10.1001/jama.271.9.703>
8. JP, S. (1992). Applied multivariate statistics for the social sciences *Hillsdale, NJ:Erlbaum*, *2nd edition*. <https://doi.org/https://doi.org/10.1111/j.1751-5823.2009.00095_13.x>
9. McHugh, M. L. (2012). Interrater reliability: the kappa statistic. *Biochem Med (Zagreb)*, *22*(3), 276-282.
10. Mehmetoglu, M., and Jakobsen, T. G. . (2016). Applied Statistics Using Stata: A Guide for the Social Sciences. *SAGE*.
11. Oliver, D., Arribas, M., Radua, J., Salazar de Pablo, G., De Micheli, A., Spada, G., . . . Fusar-Poli, P. (2022). Prognostic accuracy and clinical utility of psychometric instruments for individuals at clinical high-risk of psychosis: a systematic review and meta-analysis. *Molecular Psychiatry*. <https://doi.org/10.1038/s41380-022-01611-w>
12. Schober, P., Boer, C., & Schwarte, L. A. (2018). Correlation Coefficients: Appropriate Use and Interpretation. *Anesthesia & Analgesia*, *126*(5), 1763-1768. <https://doi.org/10.1213/ane.0000000000002864>
13. Srikesavan, C. S., Shay, B., & Szturm, T. (2015). Test-retest reliability and convergent validity of a computer based hand function test protocol in people with arthritis. *The open orthopaedics journal*, *9*, 57-67. <https://doi.org/10.2174/1874325001509010057>
14. Steenkamp, J.-B. E. M., & Baumgartner, H. (1998). Assessing Measurement Invariance in Cross-National Consumer Research. *Journal of Consumer Research*, *25*(1), 78-90. <https://EconPapers.repec.org/RePEc:oup:jconrs:v:25:y:1998:i:1:p:78-90>
15. Strauss, M. E., & Smith, G. T. (2009). Construct validity: advances in theory and methodology. *Annu Rev Clin Psychol*, *5*, 1-25. <https://doi.org/10.1146/annurev.clinpsy.032408.153639>
16. Trevethan, R. (2017). Sensitivity, Specificity, and Predictive Values: Foundations, Pliabilities, and Pitfalls in Research and Practice. *Front Public Health*, *5*, 307. <https://doi.org/10.3389/fpubh.2017.00307>
17. Vandenberg, R. J., & Lance, C. E. (2000). A Review and Synthesis of the Measurement Invariance Literature: Suggestions, Practices, and Recommendations for Organizational Research. *Organizational Research Methods*, *3*(1), 4-70. <https://doi.org/10.1177/109442810031002>
18. Viera, A. J. a. G., J.M. . (2005). Understanding Interobserver Agreement: The Kappa Statistic. *Family Medicine*, *37, 360-363*.
19. Yu, C. Y. (2002). Evaluating cutoff criteria of model fit indices for latent variable models with binary and continuous outcomes [Dissertation]. *Univeristy of California, Los Angeles, CA*. <https://doi.org/https://www.statmodel.com/download/Yudissertation.pdf>
