# Supplementary material for: Safe and valid? A systematic review of the psychometric properties of culturally adapted depression scales for use among Indigenous populations
Source: Glob Ment Health (Camb). 2023 Sep 14;10:e60. doi: 10.1017/gmh.2023.52 (PMC10579654; doi:10.1017/gmh.2023.52)
Supplement: Yang et al. supplementary material 3 — Yang et al. supplementary material [file S2054425123000523sup003.docx]

**Supplemental 3**

*Detailed results on effect of adaptation on psychometric properties*

**RESULTS**:

Four (4/31, 12.9%) studies found that removing suicidal ideation questions resulted in high reliability, cross-cultural validity, and construct validity in their adapted scales (Almeida et al., 2014; Ashaba et al., 2019; Harry & Crea, 2018; Schantz et al., 2017). Scales that removed items relating to the concept of hope also had evidence for reliability, cross-cultural validity, construct validity, specificity, and discrimination in (3/31, 9.7%) studies (Baron et al., 2017; Kilburn et al., 2018; McNamara et al., 2014).

The concept of loneliness was addressed by 3 studies, who added it to increase internal constancy and construct validity (Armenta et al., 2014; Ashaba et al., 2019; Haroz et al., 2017). Scales with added items related to somatic difficulties had good reliability, construct validity, cross-cultural validity, and incremental validity in 6 studies (Armenta et al., 2014; Ashaba et al., 2019; Gelaye et al., 2013; Haroz et al., 2017; Kilburn et al., 2018; McNamara et al., 2014). Items on anger were added by 4 studies, which resulted in strong metrics for reliability, criterion validity, incremental validity, NPV, and sensitivity (Ashaba et al., 2019; Esler et al., 2008; Hackett et al., 2019; Haroz et al., 2017).

Four studies found strong psychometric properties of scales that were modified to fit a low-literacy or rural group (Campbell et al., 2008; Gallis et al., 2018; McNamara et al., 2014; Schantz et al., 2017) and 2 studies incorporated local idioms to increase understanding (Gelaye et al., 2013; Kaaya et al., 2008). The result was high reliability, criterion validity, construct validity, sensitivity, and NPV. Changes to the protocol of administering the psychometric in 1 scale yielded high reliability, sensitivity, specificity, and NPV (Marley et al., 2017). Scales that were translated often had high reliability, criterion validity, construct validity, NPV, sensitivity, specificity, discrimination, but not PPV.

**DISCUSSION**:

There was heterogeneity in the methods taken by the studies to adapt their scale. These processes were related to the psychometric performance of the overall scales (Table 2).

### Addition or deletion of items

Fourteen studies added or removed items based on revision of linguistic equivalences and cultural relevance. Of these, 4 studies modified *suicidal ideation* questions either by removing it completely or removing the item and replacing it with 2 separate questions (Almeida et al., 2014; Ashaba et al., 2019; Harry & Crea, 2018; Schantz et al., 2017). *Hope* was another concept that was removed by 3 studies. This term was found to not be sufficiently represent positive affect to the Indigenous group (Baron et al., 2017; Kilburn et al., 2018; McNamara et al., 2014).

Through discussions, 3 studies added the Indigenous equivalence of a *loneliness* question as this was important to Indigenous representations of depression (Armenta et al., 2014; Ashaba et al., 2019; Haroz et al., 2017). *Somatic difficulties* were also found to be important to the Indigenous context and were thus incorporated the modified scales of 7 studies (Armenta et al., 2014; Ashaba et al., 2019; Gelaye et al., 2013; Haroz et al., 2017; Haroz et al., 2014; Kilburn et al., 2018; McNamara et al., 2014). Finally, *anger* was a concept that was important to accurately measuring depression amongst Indigenous peoples and was added by 4 studies (Ashaba et al., 2019; Esler et al., 2008; Hackett et al., 2019; Haroz et al., 2017).

### Simplified language

Other than modifications to achieve cultural equivalence or relevance, items were modified to fit the rural or socioeconomic context. As such, simplifying language was seen as a culturally-safe method. Four studies modified words or sentence structures that were not sensitive to a low-literacy group or rural Indigenous population; these were either removed or replaced by simpler language (Campbell et al., 2008; Gallis et al., 2018; McNamara et al., 2014; Schantz et al., 2017). To further increase understanding, 2 studies added local idioms for depression as replacements (Gelaye et al., 2013; Kaaya et al., 2008).

### Fitting the cultural context

Different modes of administering scales increased understanding and cultural sensitivity. One study achieved this by changing the scale administration process, using a visual scale and developing a culturally safe space to answer questions (Marley et al., 2017). Another way of fitting the scale to the cultural context was to translate the scale to the local Indigenous language or used an Indigenous mode of phrasing, which made the scale more understandable, accessible, and acceptable. Nineteen studies did this, which provided more accurate estimates of depression prevalence in the population (Ashaba et al., 2019; Baron et al., 2017; Bass et al., 2008; Bougie et al., 2016; Caneo et al., 2020; Chapleski et al., 1997; Denckla et al., 2017; Ekeroma et al., 2012; Esler et al., 2007; Fernandes et al., 2011; Gallis et al., 2018; Ganguli et al., 1999; Gelaye et al., 2013; Husain et al., 2006; Kilburn et al., 2018; Marley et al., 2017; Sarkar et al., 2015; Schantz et al., 2017; Tiburcio Sainz & Natera Rey, 2007).

**References**

Almeida, O. P., Flicker, L., Fenner, S., Smith, K., Hyde, Z., Atkinson, D., . . . LoGiudice, D. (2014). The Kimberley assessment of depression of older Indigenous Australians: prevalence of depressive disorders, risk factors and validation of the KICA-dep scale. *PLoS One*, *9*(4), e94983. <https://doi.org/10.1371/journal.pone.0094983>

Armenta, B. E., Sittner Hartshorn, K. J., Whitbeck, L. B., Crawford, D. M., & Hoyt, D. R. (2014). A longitudinal examination of the measurement properties and predictive utility of the Center for Epidemiologic Studies Depression Scale among North American Indigenous adolescents. *Psychol Assess*, *26*(4), 1347-1355. <https://doi.org/10.1037/a0037608>

Ashaba, S., Cooper-Vince, C., Vořechovská, D., Maling, S., Rukundo, G. Z., Akena, D., & Tsai, A. C. (2019). Development and validation of a 20-item screening scale to detect major depressive disorder among adolescents with HIV in rural Uganda: A mixed-methods study. *SSM - Population Health*, *7*, 100332. <https://doi.org/https://doi.org/10.1016/j.ssmph.2018.100332>

Baron, E. C., Davies, T., & Lund, C. (2017). Validation of the 10-item Centre for Epidemiological Studies Depression Scale (CES-D-10) in Zulu, Xhosa and Afrikaans populations in South Africa. *BMC Psychiatry*, *17*(1), 6. <https://doi.org/10.1186/s12888-016-1178-x>

Bass, J. K., Ryder, R. W., Lammers, M.-C., Mukaba, T. N., & Bolton, P. A. (2008). Post-partum depression in Kinshasa, Democratic Republic of Congo: Validation of a concept using a mixed-methods cross-cultural approach. *Tropical medicine & international health*, *13*(12), 1534-1542. <https://doi.org/10.1111/j.1365-3156.2008.02160.x>

Bougie, E., Arim, R. G., Kohen, D. E., & Findlay, L. C. (2016). Validation of the 10-item Kessler Psychological Distress Scale (K10) in the 2012 Aboriginal Peoples Survey. *Health Rep*, *27*(1), 3-10. <https://www.ncbi.nlm.nih.gov/pubmed/26788720>

Campbell, A., Hayes, B., & Buckby, B. (2008). Aboriginal and Torres Strait Islander women's experience when interacting with the Edinburgh Postnatal Depression Scale: A brief note. *The Australian journal of rural health*, *16*(3), 124-131. <https://doi.org/10.1111/j.1440-1584.2007.00930.x>

Caneo, C., Toro, P., & Ferreccio, C. (2020). Validity and Performance of the Patient Health Questionnaire (PHQ-2) for Screening of Depression in a Rural Chilean Cohort. *Community mental health journal*, *56*(7), 1284-1291. <https://doi.org/10.1007/s10597-020-00605-8>

Chapleski, E. E., Lamphere, J. K., Kaczynski, R., Lichtenberg, P. A., & Dwyer, J. W. (1997). Structure of a Depression Measure among American Indian Elders: Confirmatory Factor Analysis of the CES-D Scale. *Research on aging*, *19*(4), 462-485. <https://doi.org/10.1177/0164027597194004>

Denckla, C. A., Ndetei, D. M., Mutiso, V. N., Musyimi, C. W., Musau, A. M., Nandoya, E. S., . . . McKenzie, K. (2017). Psychometric properties of the Ndetei-Othieno-Kathuku (NOK) Scale: A mental health assessment tool for an African setting. *Journal of child and adolescent mental health*, *29*(1), 39-49. <https://doi.org/10.2989/17280583.2017.1310729>

Ekeroma, A. J., Ikenasio-Thorpe, B., Weeks, S., Kokaua, J., Puniani, K., Stone, P., & Foliaki, S. A. (2012). Validation of the Edinburgh Postnatal Depression Scale (EPDS) as a screening tool for postnatal depression in Samoan and Tongan women living in New Zealand. *The New Zealand medical journal*, *125*(1355), 41-49. <http://ovidsp.ovid.com/ovidweb.cgi?T=JS&PAGE=reference&D=med8&NEWS=N&AN=22722214>

Esler, D., Johnston, F., Thomas, D., & Davis, B. (2008). The validity of a depression screening tool modified for use with Aboriginal and Torres Strait Islander people. *Aust N Z J Public Health*, *32*(4), 317-321. <https://doi.org/10.1111/j.1753-6405.2008.00247.x>

Esler, D. M., Johnston, F., & Thomas, D. (2007). The acceptability of a depression screening tool in an urban, Aboriginal community-controlled health service. *Aust N Z J Public Health*, *31*(3), 259-263. <https://www.ncbi.nlm.nih.gov/pubmed/17679245>

Fernandes, M. C., Srinivasan, K., Stein, A. L., Menezes, G., Sumithra, R., & Ramchandani, P. G. (2011). Assessing prenatal depression in the rural developing world: a comparison of two screening measures. *Arch Womens Ment Health*, *14*(3), 209-216. <https://doi.org/10.1007/s00737-010-0190-2>

Gallis, J. A., Maselko, J., O'Donnell, K., Song, K., Saqib, K., Turner, E. L., & Sikander, S. (2018). Criterion-related validity and reliability of the Urdu version of the patient health questionnaire in a sample of community-based pregnant women in Pakistan. *PeerJ (San Francisco, CA)*, *6*, e5185-e5185. <https://doi.org/10.7717/peerj.5185>

Ganguli, M., Dube, S., Johnston, J. M., Pandav, R., Chandra, V., & Dodge, H. H. (1999). Depressive symptoms, cognitive impairment and functional impairment in a rural elderly population in India: a Hindi version of the geriatric depression scale (GDS-H). *International journal of geriatric psychiatry*, *14*(10), 807-820. <https://doi.org/10.1002/(SICI)1099-1166(199910)14:10><807::AID-GPS31>3.0.CO;2-#

Gelaye, B., Williams, M. A., Lemma, S., Deyessa, N., Bahretibeb, Y., Shibre, T., . . . Andrew Zhou, X. H. (2013). Validity of the Patient Health Questionnaire-9 for depression screening and diagnosis in East Africa. *Psychiatry Res*, *210*(2), 653-661. <https://doi.org/10.1016/j.psychres.2013.07.015>

Hackett, M. L., Teixeira‐Pinto, A., Farnbach, S., Glozier, N., Skinner, T., Askew, D. A., . . . Brown, A. (2019). Getting it Right: Validating a culturally specific screening tool for depression ( aPHQ‐9) in Aboriginal and Torres Strait Islander Australians. *Medical Journal of Australia*, *211*(1), 24-30. <https://doi.org/10.5694/mja2.50212>

Haroz, E. E., Bass, J., Lee, C., Oo, S. S., Lin, K., Kohrt, B., . . . Bolton, P. (2017). Development and cross-cultural testing of the International Depression Symptom Scale (IDSS): a measurement instrument designed to represent global presentations of depression. *Glob Ment Health (Camb)*, *4*, e17. <https://doi.org/10.1017/gmh.2017.16>

Haroz, E. E., Bass, J. K., Lee, C., Murray, L. K., Robinson, C., & Bolton, P. (2014). Adaptation and testing of psychosocial assessment instruments for cross-cultural use: an example from the Thailand Burma border. *BMC Psychology*, *2*(1), 31-31. <https://doi.org/10.1186/s40359-014-0031-6>

Harry, M. L., & Crea, T. M. (2018). Examining the measurement invariance of a modified CES-D for American Indian and non-Hispanic White adolescents and young adults. *Psychol Assess*, *30*(8), 1107-1120. <https://doi.org/10.1037/pas0000553>

Husain, N., Gater, R., Tomenson, B., & Creed, F. (2006). Comparison of the Personal Health Questionnaire and the Self Reporting Questionnaire in rural Pakistan. *J Pak Med Assoc*, *56*(8), 366-370.

Kaaya, S. F., Lee, B., Mbwambo, J. K., Smith-Fawzi, M. C., & Leshabari, M. T. (2008). Detecting Depressive Disorder With a 19-Item Local Instrument in Tanzania. *International Journal of Social Psychiatry*, *54*(1), 21-33. <https://doi.org/10.1177/0020764006075024>

Kilburn, K., Prencipe, L., Hjelm, L., Peterman, A., Handa, S., & Palermo, T. (2018). Examination of performance of the Center for Epidemiologic Studies Depression Scale Short Form 10 among African youth in poor, rural households. *BMC Psychiatry*, *18*(1), 201. <https://doi.org/10.1186/s12888-018-1774-z>

Marley, J. V., Kotz, J., Engelke, C., Williams, M., Stephen, D., Coutinho, S., & Trust, S. K. (2017). Validity and Acceptability of Kimberley Mum's Mood Scale to Screen for Perinatal Anxiety and Depression in Remote Aboriginal Health Care Settings. *PLoS One*, *12*(1), e0168969. <https://doi.org/https://dx.doi.org/10.1371/journal.pone.0168969>

McNamara, B. J., Banks, E., Gubhaju, L., Williamson, A., Joshy, G., Raphael, B., & Eades, S. J. (2014). Measuring psychological distress in older Aboriginal and Torres Strait Islanders Australians: a comparison of the K-10 and K-5. *Australian and New Zealand Journal of Public Health*, *38*(6), 567-573. <https://doi.org/10.1111/1753-6405.12271>

Sarkar, S., Kattimani, S., Roy, G., Premarajan, K. C., & Sarkar, S. (2015). Validation of the Tamil version of short form Geriatric Depression Scale-15. *Journal of neurosciences in rural practice*, *6*(3), 442-1446. <https://doi.org/10.4103/0976-3147.158800>

Schantz, K., Reighard, C., Aikens, J. E., Aruquipa, A., Pinto, B., Valverde, H., & Piette, J. D. (2017). Screening for depression in Andean Latin America: Factor structure and reliability of the CES-D short form and the PHQ-8 among Bolivian public hospital patients. *Int J Psychiatry Med*, *52*(4-6), 315-327. <https://doi.org/10.1177/0091217417738934>

Tiburcio Sainz, M., & Natera Rey, G. (2007). Adaptación al contexto ñahñú del Cuestionario de Enfrentamientos (CQ), la Escala de Síntomas (SRT) y la Escala de Depresión del Centro de Estudios Epidemiológicos (CES-D). *Salud mental (México)*, *30*(3), 48-58.
