# Supplementary material for: Safe and valid? A systematic review of the psychometric properties of culturally adapted depression scales for use among Indigenous populations
Source: Glob Ment Health (Camb). 2023 Sep 14;10:e60. doi: 10.1017/gmh.2023.52 (PMC10579654; doi:10.1017/gmh.2023.52)
Supplement: Yang et al. supplementary material 4 — Yang et al. supplementary material [file S2054425123000523sup004.docx]

**Supplemental 4**

*ROBIS Tool to assess risk of bias in systematic reviews*

# Phase 1: Assessing relevance (Optional)

ROBIS is designed to assess the risk of bias in reviews with questions relating to interventions, aetiology, diagnosis and prognosis. State your overview/guideline question (target question) and the question being addressed in the review being assessed:

What are the psychometric characteristics of culturally adapted depression scales for use with Indigenous populations?

**Intervention reviews: N/A**

| **Category** | **Target question (e.g. overview or guideline)** | **Review being assessed** |
| --- | --- | --- |
| Patients/Population(s): |  |  |
| Intervention(s): |  |  |
| Comparator(s): |  |  |
| Outcome(s): |  |  |

**For aetiology reviews: N/A**

| **Category** | **Target question (e.g. overview or guideline)** | **Review being assessed** |
| --- | --- | --- |
| Patients/Population(s): |  |  |
| Exposure(s) and comparator(s): |  |  |
| Outcome(s): |  |  |

**For Diagnosis reviews: N/A**

| **Category** | **Target question (e.g. overview or guideline)** | **Review being assessed** |
| --- | --- | --- |
| Patients): | Indigenous populations at risk for major depression | Indigenous populations with depressive symptomology |
| Index test(s): | Depression scales | Adapted depression scales |
| Reference standard: | Gold-standard depression measures | Gold-standard diagnostic instruments available in the region of testing |
| Target condition: | Major depression | Major depression |

**For prognostic reviews: N/A**

| **Category** | **Target question (e.g. overview or guideline)** | **Review being assessed** |
| --- | --- | --- |
| Patients: |  |  |
| Outcome to be predicted: |  |  |
| Intended use of model: |  |  |
| Intended moment in time: |  |  |

| Does the question addressed by the review match the target question? | YES/NO/UNCLEAR |
| --- | --- |

# Phase 2: Identifying concerns with the review process

| **DOMAIN 1: STUDY ELIGIBILITY CRITERIA** |
| --- |
| Describe the study eligibility criteria, any restrictions on eligibility and whether there was evidence that objectives and eligibility criteria were pre-specified:    Eligibility criteria:  i) the study is prospective and tested an adapted depression measure, ii) the depression measure was tested amongst an Indigenous population, iii) the study reported the methods of cultural adaptation, including processes to increase acceptability, and which modifications were made to the scale items, iv) the study reported at least one psychometric property of the scale as it was measured with the Indigenous sample (within the domains of reliability, validity, and clinical utility), v) the study reported in the English language  Exclusion criteria:  i) the adaptation process(es) were not reported, ii) the adapted scale did not measure major depression or depression constructs, iii) the study did not specify nor fulfill the specified Indigenous identify of the population, iv) the study did not report psychometric properties of the scale |
| 1.1 Did the review adhere to pre-defined objectives and eligibility criteria? Y/PY/PN/N/NI  1.2 Were the eligibility criteria appropriate for the review question? Y/PY/PN/N/NI  1.3 Were eligibility criteria unambiguous? Y/PY/PN/N/NI  1.4 Were any restrictions in eligibility criteria based on study Y/PY/PN/N/NI  characteristics appropriate (e.g. date, sample size, study quality, outcomes measured)?  1.5 Were any restrictions in eligibility criteria based on sources of Y/PY/PN/N/NI  information appropriate (e.g. publication status or format, language, availability of data)? |
| Concerns regarding specification of study eligibility criteria: LOW/HIGH/UNCLEAR  Rationale for concern:    The eligibility criteria were appropriate for the purposes of this review. This allowed the review to proceed with a clearly focused research question and objective. Specific details about the cultural adaptations were provided, including the type of original scale or what gold standard the adapted scale was measured against. However, since the prevalence of depression in the population the scales were tested in was sometimes unreported or unable to be determined, and this may have affected the ability for the study to report certain psychometric domains. Additionally, because we accepted studies that adapted scales for Indigenous peoples across the world, it is possible our search criteria did not capture studies published in another language or used terms different from what we used to screen articles. |

| **DOMAIN 2: IDENTIFICATION AND SELECTION OF STUDIES** |
| --- |
| Describe methods of study identification and selection (e.g. number of reviewers involved):    A systematic search was carried out over 6 databases that were deemed appropriate for the theme of the review: Ovid Medline, Pubmed, PsycInfo, Embase, CINAHL, and Global Health. This search strategy was checked and confirmed for accuracy by a university librarian. Two blinded reviewers selected eligible abstracts and titles for further investigation at the full-text stage. Four reviewers screened texts in a blinded manner. A third reviewer confirmed the eligibility criteria and rendered a decision on any discordance. Additionally, we searched for grey literature identified through Google scholar, open access repositories, and through citation relationships. The process is documented on a PRISMA flowchart which also lists the reasons for exclusion at each stage. The full search strategy is recorded on a table that details all search terminology, syntax, and combinations with Boolean operators. |
| 2.1 Did the search include an appropriate range of databases/electronic Y/PY/PN/N/NI  sources for published and unpublished reports?  2.2 Were methods additional to database searching used to identify  relevant reports?  Y/PY/PN/N/NI  2.3 Were the terms and structure of the search strategy likely to retrieve  as many eligible studies as possible?  Y/PY/PN/N/NI  2.4 Were restrictions based on date, publication format, or language appropriate ? Y/PY/PN/N/NI  2.5 Were efforts made to minimise error in selection of studies?  Y/PY/PN/N/NI |
| Concerns regarding methods used to identify and/or select studies: LOW/HIGH/UNCLEAR  Rationale for concern:  Databases were systematically searched using Medical Subject Headings (MeSH) heading and subheading combinations that were relevant to the concepts of the study. These terms were accurate reflections of the themes of the papers as well as the original keywords used. We found that the syntax used for different databases were appropriate. We also carried out advance searches by mapping terms to subject headings. Searching for grey literature and citation relationships supplemented our database searches; this was a method to avoid various forms of bias in what studies are ultimately included in our review. Two or more reviewers were involved in screening both titles/abstracts and full texts at each staged, blinded. Restrictions were appropriate, such as restricting to the English language in order to have uniform psychometrics which could ensure comparability between the data of the studies. |

| **DOMAIN 3: DATA COLLECTION AND STUDY APPRAISAL** |
| --- |
| Describe methods of data collection, what data were extracted from studies or collected through other means, how risk of bias was assessed (e.g. number of reviewers involved) and the tool used to assess risk of bias:    From each article, two researchers independently extracted data on characteristics of culturally adapted scales, grouping results by type of adapted scale. This included data on the scale’s clinical use, which gold standard it was measured against, the optimal culturally safe cut-off, and which population the cut-off was determined for. Additionally, 2 researchers independently reported metrics of performance of the adapted scale, grouping results by type of scale and reporting which adaptation methods were used. This included the psychometric characteristics of reliability, validity, and clinical utility. The quality of psychometric performance was evaluated and coded by 2 authors, following the guidelines on the quality criteria checklist. At any point, any discordance between the two researchers was managed through a discussion and resolved by a third researcher. |
| 3.1 Were efforts made to minimise error in data collection? Y/PY/PN/N/NI  3.2 Were sufficient study characteristics available for both review authors and readers to be able to interpret the results? Y/PY/PN/N/NI  3.3 Were all relevant study results collected for use in the synthesis? Y/PY/PN/N/NI  3.4 Was risk of bias (or methodological quality) formally assessed using appropriate criteria? Y/PY/PN/N/NI  3.5 Were efforts made to minimise error in risk of bias assessment? Y/PY/PN/N/NI |
| Concerns regarding methods used to collect data and appraise studies: LOW/HIGH/UNCLEAR  Rationale for concern:  A structed data extraction form was created prior to data collection to ensure a standardized method of data extraction between all researchers. Statistical and numerical data, and primary study characteristics of the articles were rigorously checked and rechecked several times before analysis of the results. We made sure that there was no discordance and total agreeance on the quantitative and qualitative data between all researchers involved by the end of the study appraisal phase. All data was relevant to our synthesis and interpretation of the results. There is no risk of error from statistical transformations since we did not rely on this process to derive data. However, a formal risk of bias assessment was not carried out. Most studies did not report effect sizes. Our focus was not on effect size but rather, the quality of psychometric performance across each type of adapted scale. |

| **DOMAIN 4: SYNTHESIS AND FINDINGS** |  |
| --- | --- |
| Describe synthesis methods:  We reported results on the psychometric properties displayed by scales following adaptation. The thresholds to classify the quality of psychometric performance (poor, moderate, strong) were determined through a literature search on the cut-offs most validated or commonly used in other studies, especially validations studies. This drove our analysis on patterns in psychometric performance, rather than a statistical synthesis from primary studies. We did not interpret findings in terms of effect sizes, standard errors, or standard deviations. We did not perform a statistical synthesis since this was not appropriate for our review. | |
| 4.1 Did the synthesis include all studies that it should? | Y/PY/PN/N/NI |
| 4.2 Were all pre-defined analyses reported or departures explained? | Y/PY/PN/N/NI |
| 4.3 Was the synthesis appropriate given the nature and similarity in the research questions, study designs and outcomes across included studies? | Y/PY/PN/N/NI |
| 4.4 Was between-study variation (heterogeneity) minimal or addressed in the synthesis? | Y/PY/PN/N/NI |
| 4.5 Were the findings robust, e.g. as demonstrated through funnel plot or sensitivity analyses? | Y/PY/PN/N/NI |
| 4.6 Were biases in primary studies minimal or addressed in the synthesis? | Y/PY/PN/N/NI |
| Concerns regarding the synthesis and findings: LOW/HIGH/UNCLEAR  Rationale for concern:  The synthesis included all studies known to have collected data relevant to the research questions. All studies reported the desired results of collected data. No studies were omitted due to their statistical computations. The methodology of the synthesis was driven by the nature of the studies and the questions being asked, however, since a meta-analysis was not undertaken, no statistical methods were used, such as an accepted method of statistically combining studies or a bivariate approach. As well, since this was not a meta-analysis, heterogeneity was not considered. The variation in results of the studies were not assessed. Since we did not collect data on effect sizes, we did not analyze their relationship with study sizes. The robustness of the findings was not demonstrated through a funnel plot or sensitivity analyses. | |

Y=YES, PY=PROBABLY YES, PN=PROBABLY NO, N=NO, NI=NO INFORMATION

# Phase 3: Judging risk of bias

Summarize the concerns identified during the Phase 2 assessment:

| **Domain** | **Concern** | **Rationale for concern** |
| --- | --- | --- |
| 1. Concerns regarding specification of study eligibility criteria | Low | Eligibility criteria was clear and generally unambiguous to specify the review questions and objectives. Details on eligible populations were reported. |
| 2. Concerns regarding methods used to identify and/or select studies | Low | The process of screening titles/abstracts and full-texts are reported and included multiple reviewers. It is not likely that relevant studies were missed by the searches. Search strategies were carried out systematically over all the databases and the restrictions were appropriate. |
| 3. Concerns regarding methods used to collect data and appraise studies | Low | All articles were assessed independently by two reviewers who extracted the appropriate data independently. There is a lack of formal quality assessment because effect sizes were not used (and rarely reported). There are significant study details to allow the reader to interpret the results. There is a low chance of risk of bias in the data collection and study appraisal processes. |
| 4. Concerns regarding the synthesis and findings | Unclear | The methodology of the synthesis was driven by the nature of the studies and the questions being asked, however, since a meta-analysis was not undertaken, no statistical methods were deemed necessary. Although a statistical synthesis was not appropriate, a qualitative overview of results was reported. |

| **RISK OF BIAS IN THE REVIEW** |  |
| --- | --- |
| Describe whether conclusions were supported by the evidence: |  |
| A. Did the interpretation of findings address all of the concerns identified in Domains 1 to 4? | Y/PY/PN/N/NI |
| B. Was the relevance of identified studies to the review's research question appropriately considered? | Y/PY/PN/N/NI |
| C. Did the reviewers avoid emphasizing results on the basis of their statistical significance? | Y/PY/PN/N/NI |
| Risk of bias in the review: LOW/HIGH/UNCLEAR  Rationale for risk:  Any concerns raised in Phase 2 were appropriately considered in the review conclusions. The review considers the relevance of the identified articles to the research questions. Overall, the findings of the review are likely to be reliable. | |

Y=YES, PY=PROBABLY YES, PN=PROBABLY NO, N=NO, NI=NO INFORMATION
